# K-Neighbourhood Analysis: A Method for Understanding SMLM Images as Compositions of Local Neighbourhoods

**DOI:** 10.3389/fbinf.2021.724127

**Published:** 2021-10-18

**Authors:** Kristen Feher, Matthew S. Graus, Simao Coelho, Megan V. Farrell, Jesse Goyette, Katharina Gaus

**Affiliations:** ^1^ School of Medical Sciences, EMBL Australia Node in Single Molecule Science, University of New South Wales, Kensington, NSW, Australia; ^2^ ARC Centre of Excellence in Advanced Molecular Imaging, University of New South Wales, Sydney, NSW, Australia; ^3^ Structural Biology Program, Memorial Sloan Kettering Cancer Center, New York, NY, United States

**Keywords:** TCR clustering, single molecule localisation microscopy, image analysis, point pattern analysis, clustering, local density estimation, local indicators of spatial association

## Abstract

Single molecule localisation microscopy (SMLM) is a powerful tool that has revealed the spatial arrangement of cell surface signalling proteins, producing data of enormous complexity. The complexity is partly driven by the convolution of technical and biological signal components, and partly by the challenge of pooling information across many distinct cells. To address these two particular challenges, we have devised a novel algorithm called K-neighbourhood analysis (KNA), which emphasises the fact that each image can also be viewed as a composition of local neighbourhoods. KNA is based on a novel transformation, spatial neighbourhood principal component analysis (SNPCA), which is defined by the PCA of the normalised *K*-nearest neighbour vectors of a spatially random point pattern. Here, we use KNA to define a novel visualisation of individual images, to compare within and between groups of images and to investigate the preferential patterns of phosphorylation. This methodology is also highly flexible and can be used to augment existing clustering methods by providing clustering diagnostics as well as revealing substructure within microclusters. In summary, we have presented a highly flexible analysis tool that presents new conceptual possibilities in the analysis of SMLM images.

## Introduction

SMLM has given insight into the spatial arrangement of signalling proteins with unprecedented resolution (Huang et al., 2009; Patterson et al., 2010; Nicovich et al., 2017; Schnitzbauer et al., 2017). The complex spatial arrangement of these proteins is an emergent property of interactions between many types of proteins, and reflect the external environment that is being sensed, as well as being dependent on prior states of the cell (Ditlev et al., 2018; Yoo et al., 2019; Espinosa et al., 2020). Being a snapshot of a dynamic process, it can be expected that the image will contain a mixture of spatially localised subprocesses that coexist side by side. Similar processes that are spatially separated may not be exactly temporally synchronised. For example, protein clustering, i.e., transition away from well mixed homogenous states towards droplets/condensates and beyond, is dynamic and may be at different stages of progress in different parts of the cell, or even within larger condensates. While genetically identical cells grown under the same conditions will result in unique and random instantiations, it could be expected that they are linked by universal properties, e.g., distribution of cluster sizes or distribution of distances between clusters.

Within the field of T cell activation, SMLM imaging has made a large impact on our understanding of the underlying processes. It is now well established that spatial organisation of T cell receptor (TCR) in the plasma membrane determines the probability of phosphorylation and downstream signalling processes (Grakoui et al., 1999; Pageon et al., 2016a; Sherman et al., 2016). As proteins that reside in the same regions in the plasma membrane are more likely to interact, it is important to map and better understand the organizing principles of membrane proteins (Saka et al., 2014). For example, chimeric antigen receptors (CARs) need to integrate into the T cell signalling network (Lim and June 2017) and therefore ought to adopt a spatial organisation similar to that of the TCR. However, quantifying the diversity in spatial organisations that a single protein can adopt within and across individual cells has remained challenging. The TCR, for example, has been described as randomly distributed (Rossboth et al., 2018), monomeric (James et al., 2007) or as a functional dimer (Kuhns et al., 2010), to form pre-existing (Sherman et al., 2011) (Schamel et al., 2005; Kumar et al., 2011) and antigen-induced clusters (Kumar et al., 2011; Pageon et al., 2016a; Boniface et al., 1998), which can reside in large, immobile protein islands (Lillemeier et al., 2010; Drbal et al., 2007; Purbhoo et al., 2010).

The mode of imaging adds a further layer of complexity, as the molecules are not directly observed. When individual molecules are not spatially resolvable due to the diffraction limit, sparsity is induced in both space and time by stochastic photoactivation or binding of fluorescent probes that generate localisations. Localisations are points in ${\mathbb{R}}^{2}$ (for 2D imaging, or ${\mathbb{R}}^{3}$ for 3D imaging), and a set of localisations over a small area is evidence for the existence of a molecule. In real images, it is virtually impossible to attribute localisations to specific molecular numbers and positions with high confidence (Feher et al., 2019). Molecules may be tightly packed and thus sets of localisations arising from multiple molecules may be spatially overlapping, dependent on instrument precision. Labelling efficiency and stochastic blinking effects induce a fundamental limitation in molecular counting at individual protein sites. It is possible to estimate the underlying molecular positions by collapsing repeated localisations in a procedure called “grouping” but this can introduce new artefacts due to the afore-mentioned reasons.

Overall, it can be expected that SMLM images of cells have a complex multiscale structure, generated by convoluting biological with technical effects and overlaid with spurious noise localisations. For any given localisation, the spatial arrangement of the immediately neighbouring localisations is dominated by the photophysics and the distance of the nearest neighbouring molecule. The spatial arrangement of more distant localisations is influenced by the emergent properties of many interacting proteins. To date, SMLM images of one or two protein types are possible, but technical advances in simultaneously imaging multiple protein types are underway. The broad goal of SMLM data analysis is to extract instances or types of protein arrangements and link it to biological function. Examples include droplet size and composition, indicating previous recruitment of proteins to the site; or proximity of proteins types to each other, indicating the possibility or otherwise of biochemical reactions taking place.

General approaches to analysing point pattern data often involve clustering or density estimation. Examples within SMLM data analysis include Ripley’s K-function (Owen et al., 2010), pair correlation (Sengupta et al., 2013; Shivanandan et al., 2016), density-based clustering (Pageon et al., 2016a; Jiang et al., 2017; Rubin-Delanchy et al., 2015) or tessellation based analysis (Levet et al., 2015). Clustering is most straightforward when spacing between clusters (inter-cluster) dominates the spacing between points within the cluster (intra-cluster) in all instances, and there are no noise points between the clusters. In this case, a single unambiguous clustering can generally be found. As intra-cluster spacing grows with respect to inter-cluster spacing and background noise increases, multiple cluster organisations could be obtained depending on the chosen optimisation criteria (Liu et al., 2015; Maurus and Plant, 2016). On the other hand, density estimates are highly dependent on the chosen bandwidth (Davies and Baddeley, 2018), and thus multiple bandwidths may be needed to fully describe multiscale structure. As density is an average quantity within a window, it can be problematic to describe discontinuous events, e.g., a small cluster surrounded by a relatively large empty space.

Bridging the gap between clustering and density estimation are local indicators of spatial association (LISA) methods (Anselin, 1995). They represent the contribution of each point to a global spatial statistic, reflecting local spatial arrangements. For example, Ripley’s K-function can be decomposed into local K-functions for each localisation. Other methods aim to deconvolute the superposition of two independent point patterns (Byers and Raftery, 1998; Cressie and Collins, 2001; Redenbach et al., 2015). In this work, we describe a novel LISA-like method of characterising SMLM images that is based on a vector of nearest neighbour distances corresponding to each localisation in the image. In more detail, our work extends ideas in Byers & Raftery (Byers and Raftery, 1998), by considering the joint distribution of *K*th nearest neighbour (NN) distances (NND) for *K* = 1 … 100. This leads us to consider SMLM localisations as points in a multivariate coordinate system defined by the NNDs for each *K*, so that we can aggregate localisations with similar properties, in a manner analogous to that of Cressie and Collins (2001). However, instead of using local K-functions which requires the scale to be fixed, the NND vectors can probe the local topology of each localisation, regardless of that localisation’s local density (*K*-neighbourhood analysis).

While clustering will remain central to SMLM data analysis, we aim to expand the conceptual possibilities in a manner that does not require explicit spatial segmentation *via* clustering. Instead, we wish to view each image as a collection of local neighbourhoods and use this concept to dissect individual images and compare between entire images with minimal assumptions. This will facilitate novel visualisations of SMLM images, comparisons amongst sets of SMLM images and provide a rational framework to find associations between the spatial structures of different types of proteins. This novel method can be performed on the entire set of localisations, thereby avoiding any artefacts introduced by grouping.

The major contribution of this paper is the Spatial Neighbourhood PCA (SNPCA). This is a transformation that is derived from the normalised nearest neighbour distance vectors of each point in a spatially random point pattern, using the first *K* neighbours. This basis can be used to compress the *K*-nearest neighbour vectors of an arbitrary point pattern and compare the neighbourhood compositions between sets of arbitrary point patterns. The properties of SNPCA are investigated using simulated point patterns to show it can capture structural nuances that are not apparent with univariate measures such as local density. The SNPCA is then used to develop a novel visualisation technique for individual SMLM images. Next, a set of SMLM images of activated T cells are analysed to demonstrate the global differences of the CD3 spatial patterns that occur between different types of cells. Finally, these results are used for a downstream analysis of CD3 phosphorylation patterns.

## Materials and Methods

### Cell Culture

Jurkat-ILA1 T cells and Jurkat 76T cells were cultured in RPMI 1640 (Life Technologies, 21870076) supplemented with 10% FBS, 2 mMl-glutamine, 1 mM penicillin and streptomycin (all from Life Technologies). Characterization of the ILA1 TCR is described in the methods section of Pageon et al. (Pageon et al., 2016b). Jurkat 76 cells were transduced to express either LaG17-CD3ζ or LaG17-CD28-CD3ζ CAR construct.

### Constructs

Lentiviral anti-GFP CAR constructs were produced and transduced into Jurkat 76 cells as described in Denham et al (Denham et al., 2019). We used Jurkat 76 cells since these cells lack surface expression of the TCR complex and thus anti-CD3ζ staining was specific for CAR constructs. For bacterial expression of CAR ligand, a construct of an N-terminally Avitag-labelled monovalent (A206K mutant), dark (Y66S mutant) EGFP (Avi-dGFP) was cloned into pTRC-HisA between the NheI and HindIII restriction sites. For the PI3K PAINT probe, amino acids 322–724 (constituting the tandem Src homology two domains) of the regulatory subunit, p85, with M479S, I493S, Y504S, Y508S hydrophobic to hydrophilic mutations of residues in the interface with the catalytic domain were fused with mNeon Green on C-terminus and cloned into pET21 between the NdeI and NotI restriction sites (p85 tSH2-mNG).

### CAR Ligand and PI3K PAINT Probe Production

Chemically competent BL21 (DE3) *E. coli* cells (Agilent Technologies) were transformed with Avi-dGFP or p85 tSH2-mNG and grown on ampicillin (50 μg/ml) LB agar plates overnight at 37°C. The following day an individual colony was inoculated into LB media with 50 μg/ml ampicillin and grown in a shaker incubator overnight at 37°C. Ten ml of this starter culture was then inoculated into 1 L of LB media and the cells were grown in a shaker incubator at 37°C until the optical density at 600 nm was 0.6. The temperature in then decreased to 18°C and IPTG to 0.5 mM was added. For Avi-GFP biotin to 20 µM (to drive biotinylation of the Avitag) was added to the culture media. The protein was left to induce overnight, after which the cells were pelleted by centrifugation and stored at −80°C until protein extraction and purification was performed. Protein was extracted by thawing cells, resuspending in 50 mM NaH_2_PO_4_, 300 mM NaCl pH 7.5 (2×PBS), sonicating, and pelleting debris by centrifugation at 15,000 rcf for 15 min. The clarified lysate was passed through 2 ml Nickel-NTA agarose resin in a gravity-fed column, which was then washed with 10 column volumes of 2×PBS, then with 10 ml of 2×PBS with 10 mm imidazole. Proteins were eluted with 150 mM imidazole pH 7.5. Eluate was concentrated to 0.5 ml with a 30 kDa spin concentrator (Amicon) and a final polishing step of size exclusion chromatography on a HiPrep 16/60 Sephacryl S-200 HR (GE Healthcare) equilibrated in PBS with 1 mM DTT was performed. Purified protein was mixed with glycerol to a final concentration of 10% (v/v) and aliquots were frozen at −80°C until used.

### Bilayer Preparation

Glass coverslips were cleaned with 1M KOH, rinsed in MilliQ water, and then placed in 100% ethanol prior to plasma cleaning. Eight-well silicone chambers (Ibidi, 80841) were then attached to the plasma cleaned coverslip. A 1 mg/ml liposome solution with a lipid ratio of 96.5% DOPC (1,2-dioleoyl-*sn*-glycero-3-phosphocholine), 2% DGS-NTA(Ni) (1,2-dioleoyl-*sn*-glycero-3-{[N-(5-amino-1-carboxypentyl)iminodiacetic acid]succinyl} (nickel salt)), 1% Biotinyl-Cap-PE [1,2-dioleoyl-*sn*-glycero-3-phosphoethanolamine-N-(cap biotinyl) (sodium salt)], and 0.5% PEG5000-PE {1,2-distearoyl-*sn*-glycero-3-phosphoethanolamine-N-[methoxy (polyethylene glycol)-5000]} (ammonium salt) (mol%; all available from Avanti Polar Lipids (DOPC, 850375C) [DGS-NTA(Ni), 790404C] (Biotinyl-Cap-PE, 870273C), (PEG5000-PE, 880220C) was created by vesicle extrusion, as described previously (Beemiller et al., 2012). The lipid solution was added to each well at a 1:5 ratio with MilliQ water along with 10 mM of CaCl_2_ for 15 min and then washed three times with phosphate-buffered saline (PBS). 0.5 mM EDTA in MilliQ water was added to remove the excess CaCl_2_ followed by washing with PBS. 1 mM of NiCl_2_ in PBS was added for 15 min to recharge the NTA groups, then surfaces washed three times with PBS. Disruption of the lipid bilayer was avoided by maintaining 100–150 µl of PBS in the wells at all times.

To decorate the bilayer with proteins, 100 μg/ml of streptavidin (Life Technologies, SNN1001) and 200 ng/ml of His-tagged ICAM-1 (Thermo Fisher Scientific, 10346H08H50) were combined in PBS and added to the well for 15 min at room temperature and then washed with PBS. Biotinylated proteins were then combined with 5% BSA/PBS and added to each well for 30 min at room temperature; for Jurkat-ILA cells 1:500 dilution of pMHC 3G (from a 1 mg/ml stock) was used and for CAR-expressing Jurkat 76 cells 10 nM of dark GFP and 90 nM of dark mCherry were combined prior to being added to the bilayer. The wells are then washed with PBS to remove any unbound proteins.

### Antibody Conjugation

CF568-succinimidyl-ester (Biotium, 92131) was conjugated to soluble pCD3ζ (pY142) antibody (BD Pharmingen, 558402). CF568-succinimidyl-ester and the antibody were mixed at a 6:1 molecular at pH 8.0 for 1 h at room temperature in the dark. The antibody was purified by using Zeba desalting columns (Thermo Fisher Scientific, 89883). Absorption spectroscopy determined that the degree of labelling was 1.5:1 dye:antibody ratio.

### T Cell Activation on Bilayer and Immunostaining

The wells containing bilayers were washed with RPMI culture media and warmed to 37°C for 30 min prior to adding the cells. Cells were added to the bilayer at a density of 250,000 cells/well for 4–5 min at 37°C and fixed using 4% PFA in PBS warmed to 37°C. Fixation of cells was done for 15 min at 37°C. Prior to immunostaining, cells were permeabilized with Triton X-100 (Sigma-Aldrich, T8787) at 0.1% for 5 min at room temperature and washed with PBS. The cells were blocked with 5% BSA in PBS for 1 h at room temperature.

Staining of the cells was performed sequentially with primary labelled antibodies against l CD3ζ conjugated to Alexa Flour 647 (Abcam, 197037) and pCD3ζ (pY142) conjugated to CF568. Staining was done in 5% BSA in PBS at a concentration of 10 μg/ml for both antibodies for 30 min at room temperature in the dark, then washed with PBS. Fiducials in the form of TetraSpeck Microspheres (Thermo Fisher Scientific, T7279) were added to each well for 15 min then washed with PBS.

### dSTORM and PAINT Imaging

For dSTORM imaging, a buffer consisting of 50 mM Tris, 10% (wt/vol) glucose, 10 mM NaCl, 20 μg/ml catalase (Sigma-Aldrich, C100), 0.8 mg/ml glucose oxidase (Sigma-Aldrich, G2133), and 30 mM cysteamine (Sigma-Aldrich, 30070), pH 8.0 was used during data acquisition. Data was acquired on a Zeiss ELYRA microscope with TIRF illumination using a ×100 oil-immersion objective (NA = 1.46) coupled to a cooled, electron-multiplying charge-coupled device camera (Andor, iXon DU-897). Sample excitation was done with 637 nm laser and 561 nm laser. For single channel acquisitions 20,000 frames were collected at an exposure time of 33 ms. Sequential imaging of the fluorescent probes was performed to acquire two-channel data with the farther red channel acquired first. For each channel, 20,000 frames were collected with an exposure time of 33 ms. Drift correction and channel alignment algorithms were performed on the raw data to produce data tables containing x-y localization coordinates using Zen 2012 SP5 (Zeiss MicroImaging).

PBS buffer containing 1% (wt/vol) BSA, 0.1% (wt/vol) saponin, 1 mM DTT and 1 mM EDTA was used for the preparation and imaging of the PI3K probe. PAINT imaging was performed by adding 600 pM of the PI3K-mNeonGreen probe to the well and exciting with 488 nm laser in TIRF mode. For each cell, 10,000 frames were collected with an exposure time of 200 ms. Raw image stacks were fitted for molecular localisations and drift corrected using the “Picasso” software package (Schnitzbauer et al., 2017).

**DNA origami rulers**. A single well of an eight-well chamber (ibidi 80841) was attached to a clean coverslip and washed with 500 μl of PBS. The well was incubated with 200 μl of BSA-biotin solution (1 mg/ml in PBS) for 5 min. Excess BSA-biotin was removed by washing with 500 μl of PBS. The surface was incubated with 200 μl of neutravidin (1 mg/ml in PBS) for 5 min and washed with a PBS with 10 mM magnesium. Biotin-coated polystyrene beads (Spherotech, TP-305) (40 μg/ml) were incubated for 1 h and the excess beads were removed. The well was incubated with the DNA-origami ruler (GATTA-PAINT, HiRes 20R or 80R) diluted 40 times in PBS with 10 mM magnesium to get ∼100 rulers per field-of-view. Excess DNA origamis were removed by washing with PBS with 10 mM magnesium. The imaging strand was a 9-bp complementary target strand with Atto 655, with a concentration of 5 nM. Acquisition was performed as previously described by Coelho et al., 2020 (Coelho et al., 2020).

### Statistical Description of Localisations

Let there be N localisations in a region of interest (ROI), and each localisation is indexed by *i* with 1 ≤ *i* ≤ *N*. In order to describe each localisation *i via* its local topology, i.e., the spatial arrangement of its neighbouring localisations, the distance *D*_*ij*_ from the *i*th localisation to its *j*th nearest neighbour (NN) is calculated for 1 ≤ *j* ≤ *K*. Thus localisation *i* is described by a *K*-dimensional nearest neighbour vector *NNV*_*i*_ = (*D*_*i1*_, … , *D*_*ij*_, … , *D*_*iK*_) (*K*-neighbourhood).

### Choice of *K*

The parameter *K* is chosen to be larger than the number of localisations arising from a single molecule. However, the *K*-neighbourhood analysis is quite robust over a range of *K* values. Within large aggregates of molecules (e.g., microclusters), *K*-neighbourhoods may appear to be spatially random because the edge of the cluster has not been reached. To understand how the *K*-neighbourhoods are related to long-range structure, we examined the NNDs for *K* = 200, 500.

### Comparison of Image Localisations to Spatially Random Localisations

The expected value of *D*_*ij*_ under complete spatial randomness is *α*√*j* where *α* is a constant that accounts for the rate of the Poisson process, i.e., the density of localisations (Thompson, 1956). To examine a localisation’s topology independently of scale, we set *α* = 1/√*K* and normalised *NNV*_*i*_ to yield *nNNV*_*i*_ *= NNV*_*i*_ */D*_*iK*_ such that *nNNV*_*i*_ falls on the interval (0, 1). Additionally, the *nNNV*_*i*_s of a ROI collectively forms the rows of a table of dimension *N* × *K*, which we named NN feature table (NNFT). The NNFT is an abstract description of a ROI.

### Principal Component Analysis (PCA) of the Nearest Neighbour Feature Table (NNFT)

**FIGURE 1:**
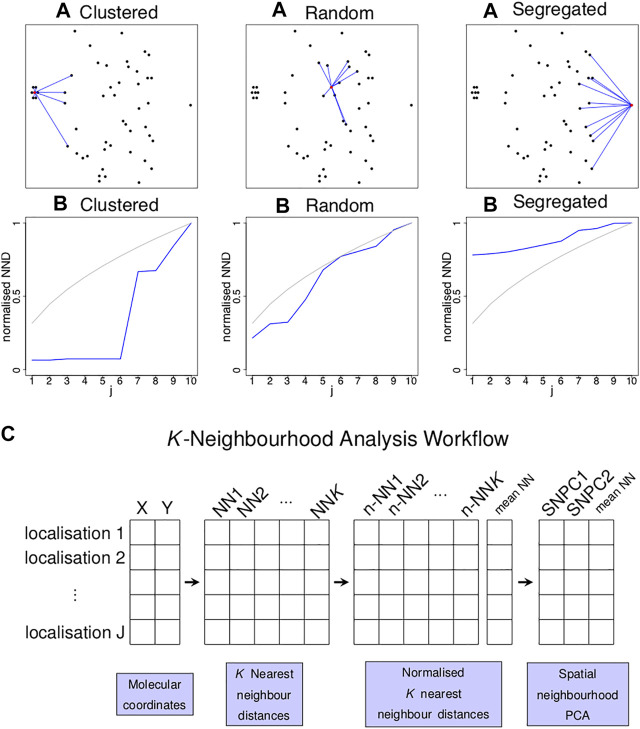
Concept of k nearest neighbour distances to identify localised spatial organisations for each point in an image. **(A)** Three examples of a single point (highlighted in red) residing in a cluster of six points **(left)**, random point distribution **(middle)** and segregated from other points **(left)** and its *K* = 10 nearest neighbours (blue lines). **(B)** Plots of the normalised distance from the red localisation to the *j*th nearest neighbour (*y*-axis) against *j* (*x*-axis) for the examples above (blue lines) and for completely spatially random (CSR) data (grey lines). Each of the three scenarios shown in **(A)** gives rise to a characteristic curve in **(B)**. **(C)** Schematic diagram illustrating the workflow of the *K*-Neighbourhood Analysis.

Localisation i can be viewed as a point in ℝ^*K*^ *via NNV*_*i*_. To summarise the major features of the NNFT (Figure 1), dimension reduction is needed. Principal component analysis (PCA) could performed on individual NNFTs to yield a new orthonormal basis corresponding to the directions of largest variance. However, each PCA will yield a different basis, reflecting the individual properties of each NNFT, rendering comparisons between images or cellular conditions impossible. To generate a universal basis for any NNFT, we simulated 100,000 completely spatial random (CSR) points with a two-dimensional Poisson process on the unit square with intensity *λ* = 10^5^. For each simulated point, the *nNNV*_*i*_ was calculated, the edge points discarded, the NNFT mean-centred and the PCA calculated. For a NNFT arising from an experimental ROI, each localisation can be represented by the orthogonal projection of its *nNNV*_*i*_ on the first two principal components of the CSR PCA, yielding new coordinates (*PC1*_*i*_, *PC2*_*i*_).

### Local Density

In each K-neighbourhood, localisation density is defined as *Den*_*i*_ = (*K*/2)/(π*D̄*_*i*_^2^), with *D̄*_*i*_ = (*1/K*)∑_*j*_ *D*_*ij*_ (i.e., the mean NND). *Den*_*i*_ is plotted on a logarithmic scale.

### Comparison of Images and Standardised Frequency Table

A group of localisations in a single image can be represented by an *N* × 3 table with the *i*th row defined (*Den*_*i*_ *, PC1*_*i*_, *PC2*_*i*_). To make comparisons between images, we compared the joint density of these three parameters. To do this, we binned each parameter into equally sized intervals, with the intervals being fixed for all images. To construct the bins, for each parameter the combined range over all images was determined. The range was then split into 50 equal sized bins, and the 1-dimensional bins were merged to form a 3-dimensional grid composed of 3-dimensional bins. Finally, the localisations were counted in each 3-dimensional bin, and the bins are clustered using average linkage hierarchical clustering (Ward, 1963) to yield a small number of 3-dimensional bin subsets (termed “groups”) that correspond to a colour key. When performed on an ensemble of images, a frequency table was obtained, with each row being one image and each column corresponding to the total frequency of localisations falling in a group of 3-dimensional bins. The frequency table is compositional, i.e., rows sum to one, so log ratio analysis is performed for dimension reduction (Aitchison and Greenacre, 2002). This entails log transformation followed by mean-centering, then principal component analysis (PCA) on the column centred matrix. To facilitate visualisation of the three categories, between component analysis is performed on the dimension reduced table, which is the PCA on the experimental means of the 3 cell types followed by the projection of the individuals onto the found space (Thioulouse, 2011). The table can also be visualised as a heatmap (Gu et al., 2016). To test for differences in variance between the experimental groups, the procedure in Anderson (Anderson, 2006) is used. To test for differences in multivariate means between the experimental groups, the procedure in Ellis et al. (Burchett et al., 2017) is used.

### Simulations

Point patterns were simulated to investigate the performance of *K*-neighbourhood analysis. Spatially random cluster centres were simulated using a Poisson process. They were populated with localisations by randomly selecting the localisation count *C* on an interval, and drawing *C* points from a bivariate normal distribution with a fixed variance and zero correlation. Finally, the simulated image is overlaid with spatially random noise localisations that are simulated with a Poisson process.

### Phosphorylation Enrichment Score

To calculate a spatially dependent phosphorylation enrichment score, each CD3ζ localisation in an image is scored TRUE or FALSE according to whether it is within 10 nm of a pCD3ζ localisation (co-localised CD3ζ localisation). For each localisation group indexed by *k* (here 1 ≤ *k* ≤ 9), the frequency of localisations in *k* that also score TRUE is compared to the frequency of all localisations in *k* such that *Enrichment* = log[*Freq(k AND TRUE)/Freq(k)*]. When *Enrichment* > 0, phosphorylation is overrepresented among localisations in *k* and when *Enrichment* < 0, phosphorylation in underrepresented among localisations in *k*. Differences of multivariate enrichment scores between experimental groups are tested as described above in “Comparison between images.” The enrichment score is additionally plotted for thresholds of 10, 20, 30, 500 nm. To understand whether enrichment scores are significantly different from zero, random scores are simulated as follows: For each image, if there are *n*_*p*_ co-localised CD3ζ localisations, then a random vector is drawn from a multinomial distribution characterised by the CD3ζ frequency vector of that image and *n*_*p*_, the random vector is normalised to sum to one, and an enrichment score is calculated. This is repeated 10,000 times, and the 5 and 95th percentiles of the simulated random scores are obtained. After repeating for each image, the minimum and maximum (respectively) scores over all images are reported and plotted.

## Results

In order to characterise the neighbourhood of any point in a point pattern (a point pattern here is defined as a set of points in ${\mathbb{R}}^{2}$ ), the vector of the first *K* nearest neighbour (NN) distances is considered. The normalised NN (n-NN) distances can be plotted as a curve vs the index *j* (for 1 ≤ *j* ≤ *K*) (Figure 1). Independently of the magnitude of the NN distances, the shape of this n-NN curve is related to the spatial organisation of neighbouring points. The n-NN curve can be compressed using a principal component analysis (PCA) that is defined using n-NN curves arising from spatially random data (SNPCA), meaning that different point patterns can be expressed using a set of common basis vectors and compared directly. The algorithm takes a point pattern and *K* as input, calculates a n-NN curve for each point and transforms it using the SNPCA. Two components have been shown to be sufficient for SNPCA (Supplementary Figures 1–3). For completeness, the mean NN value can also be stored along with the two components, so that information about the NN magnitude is available. For ease of interpretation, the mean NN value can be converted to a density. The algorithm is referred to as *K*-neighbourhood analysis (KNA).

**FIGURE 2:**
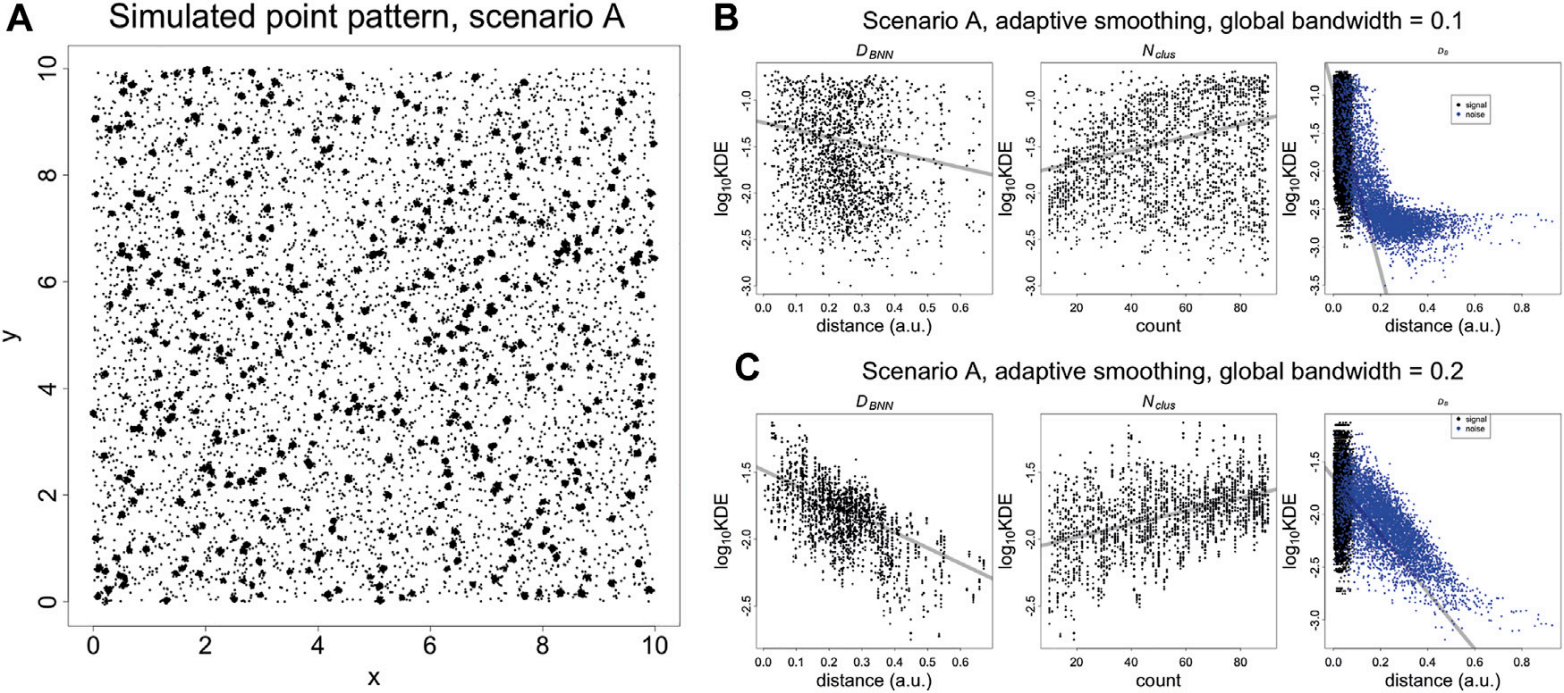
**(A)** Simulated SMLM image of spatially random molecules. Molecules are generated using a Poisson process with intensity of five over a square window with sides of length 10 (arbitrary units). The molecules are replaced with clusters of localisations, generated with a bivariate normal distribution with variance of 0.000625 and covariance of zero. The number of localisations per cluster is an integer randomly sampled on the interval (10, 90). Finally, the noise localisations are generated using a Poisson process with intensity of 50. *D*_*B*_ *, D*_*BNN*_ *, N*_*clus*_ plotted vs Kernel Density Estimates (KDE). **(B)** KDE (adaptive smoothing) of Figure 4 fitted with a global bandwidth of 0.1. **(C)** KDE (adaptive smoothing) of Figure 4 fitted with a global bandwidth of 0.2. At low values of the bandwidth, it is possible to differentiate between signal and noise localisations but the trend with *D*_*BNN*_ and *N*_*clus*_ becomes less clear. At a higher value of the bandwidth, the trend *D*_*BNN*_ and *N*_*clus*_ becomes clear but it is no longer possible to differentiate between signal and noise localisations. This highlights that a KDE at using a single bandwidth is not adequate to capture the entire spatial structure. Trend lines are fitted with a linear model and are supplied for visualisation purposes.

The properties of the KNA are investigated using simulated point patterns that mimic an SMLM image of spatially random binding sites (Figure 2A). First, the region of interest (ROI) is populated with molecules using a Poisson process of a given intensity. Next, each molecule is replaced with clusters of localisations generated from a bivariate normal distribution (fixed variance in x and y, and correlation coefficient of zero), with the number *N* of localisations being uniformly distributed between 10 and 90. Finally, spurious background localisations are generated using a Poisson process of a given intensity. For each localisation associated with a “parent” molecule (signal localisations), its local neighbourhood is strongly influenced by the total number *N*_*clus*_ of localisations associated with the parent molecule, the distance *D*_*B*_ to the parent molecule, and the distance *D*_*BNN*_ from its parent molecule to the neighbouring molecule. For noise localisations, its local neighbourhood is strongly influenced by the distance *D*_*B*_ to the nearest molecule. Further properties such as distance to the second neighbouring molecule also influence local neighbourhoods but they are not considered here.

These three quantities are plotted against kernel density estimates (adaptive smoothing using a Gaussian kernel) calculated at each point, for two different bandwidths (Figures 2B,C). For signal localisations, there are negative and positive trends (resp.) with *D*_*BNN*_ and *N*_*clus*_. However, there are two separate trends with *D*_*N*_, with density estimates occuring in the same range for signal and some noise localisations. While this differentiation can be improved by lowering the bandwidth, it indicates that a single univariate parameter such as density cannot capture the nuance of coexisting spatial arrangements. A similar phenomenon occurs when plotting nearest neighbour distances for individual values of *K* (Supplementary Figure 4).

**FIGURE 3:**
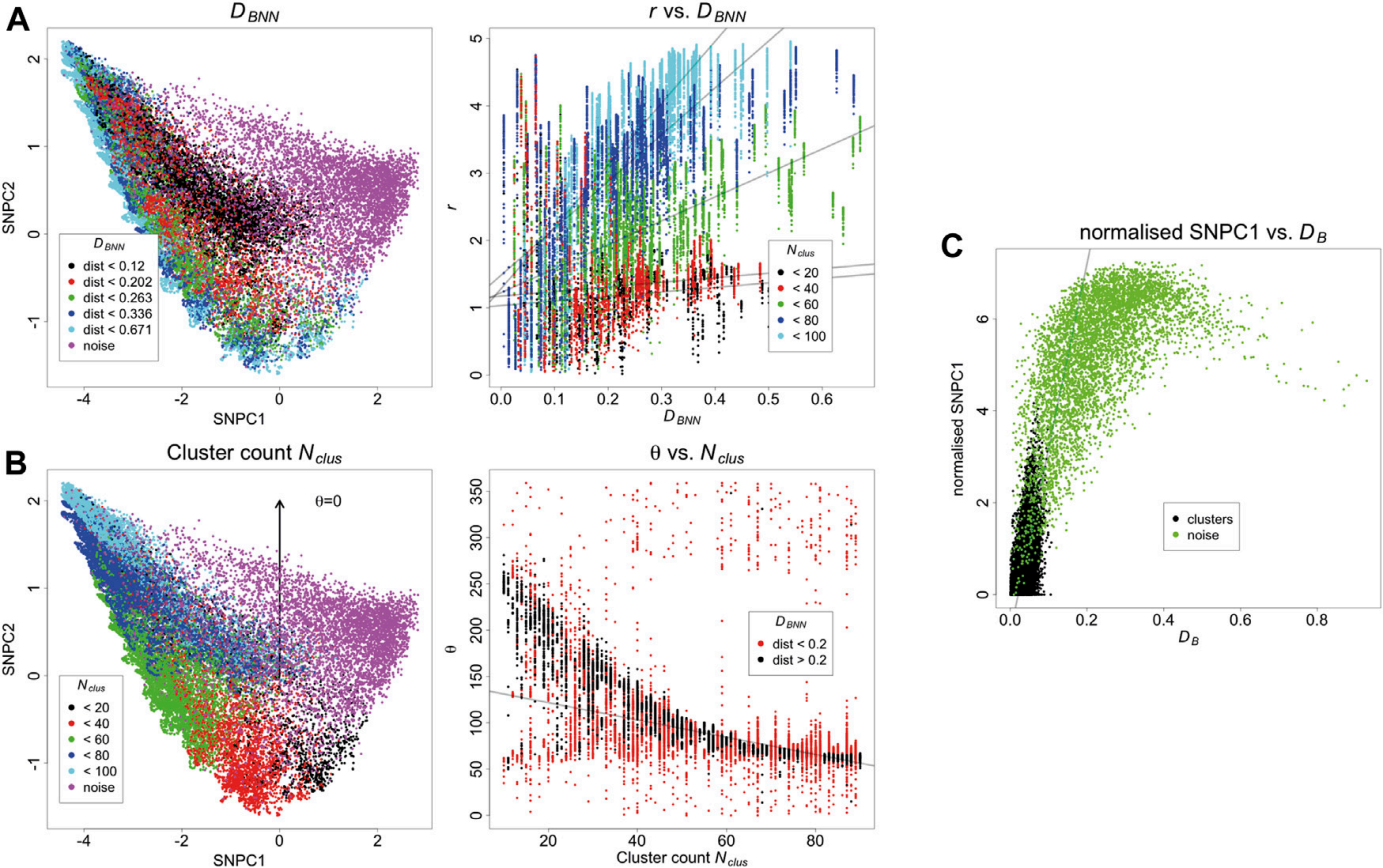
**(A)** Relationship between SNPCA and *D*_*BNN*_. *D*_*BNN*_ has a positive trend with r, and this effect is strongest for larger clusters. Trend lines are fitted with robust linear models and are supplied for visualisation purposes. **(B)** Relationship between SNPCA and cluster count *N*_*clus*_. *N*_*clus*_ has an angular dependence within the SNPCA. The relationship is strongest for well separated binding sites otherwise individual clusters appear to be larger aggregates. Trend lines are fitted with robust linear models and are supplied for visualisation purposes. **(C)** Relationship of *D*_*B*_ with SNPCA. After normalising SNPC1 to the smallest value in each cluster, it has a positive trend with *D*_*B*_. Trend lines are fitted with robust linear models and are supplied for visualisation purposes.

The KNA of the same simulated dataset is carried out, and the first two components are converted to polar coordinates *r* and *θ* relative to the origin of the axes. There is a trend between *D*_*BNN*_ and *r*, showing that *D*_*BNN*_ has radial dependence, and the slope of this trend also depends on *N*_*clus*_ (Figures 3A,B). There is a trend between *N*_*clus*_ and θ, showing that *N*_*clus*_ has an angular dependence, and this effect is strongest for clusters that are further from other clusters (as clusters in close proximity to each other start to resemble larger aggregates) (Figures 3C,D). Noise localisations occupy a distinct angular region in relation to signal localisations (Supplementary Figure 5). For localisations belonging to the same cluster, those closer to the parent molecule have a lower value of SNPC1 than those farther from the parent molecule, i.e., there is a local trend between *D*_*B*_ and SNPC1. Noise localisations generally have higher values of SNPC1 than signal localisations (Figure 3E). While these trends have been evaluated separately, they are in fact linked and jointly contribute to each localisation’s local neighbourhood. Other aspects of structure such as distance to the second nearest binding site have not been considered, but they will also potentially contribute to each local neighbourhood. Finally, as the density of noise localisations increases (Supplementary Figures 6–7), it will cause an apparent increase in the counts per cluster, and cause low count clusters to be indistinguishable from noise localisations. For this reason, meaningful comparisons can only be made between images acquired under the same imaging conditions, unless batch effects are being assessed.

Because the SNPCA is fixed, any point in the SNPC1-SNPC2 plane will always correspond to a fixed normalised NN curve, but interpretation of the process it arises from requires careful consideration of the context. For example, consider two different scenarios with different densities of noise. In scenario A (Figure 3), the density is low, and so the noise localisations in fact appear to be segregated. In other words, the definition of noise arises due to the fact they do not carry biological signal but they are not spatially random within their neighbourhoods due to positioning of surrounding clusters. In contrast, the noise points in scenario D (Supplementary Figures 6–7) are much denser and so their neighbourhoods have a larger tendency towards being spatially random. Correspondingly, their SNPC1 and SNPC2 values have decreased compared to scenario A.

**FIGURE 4:**
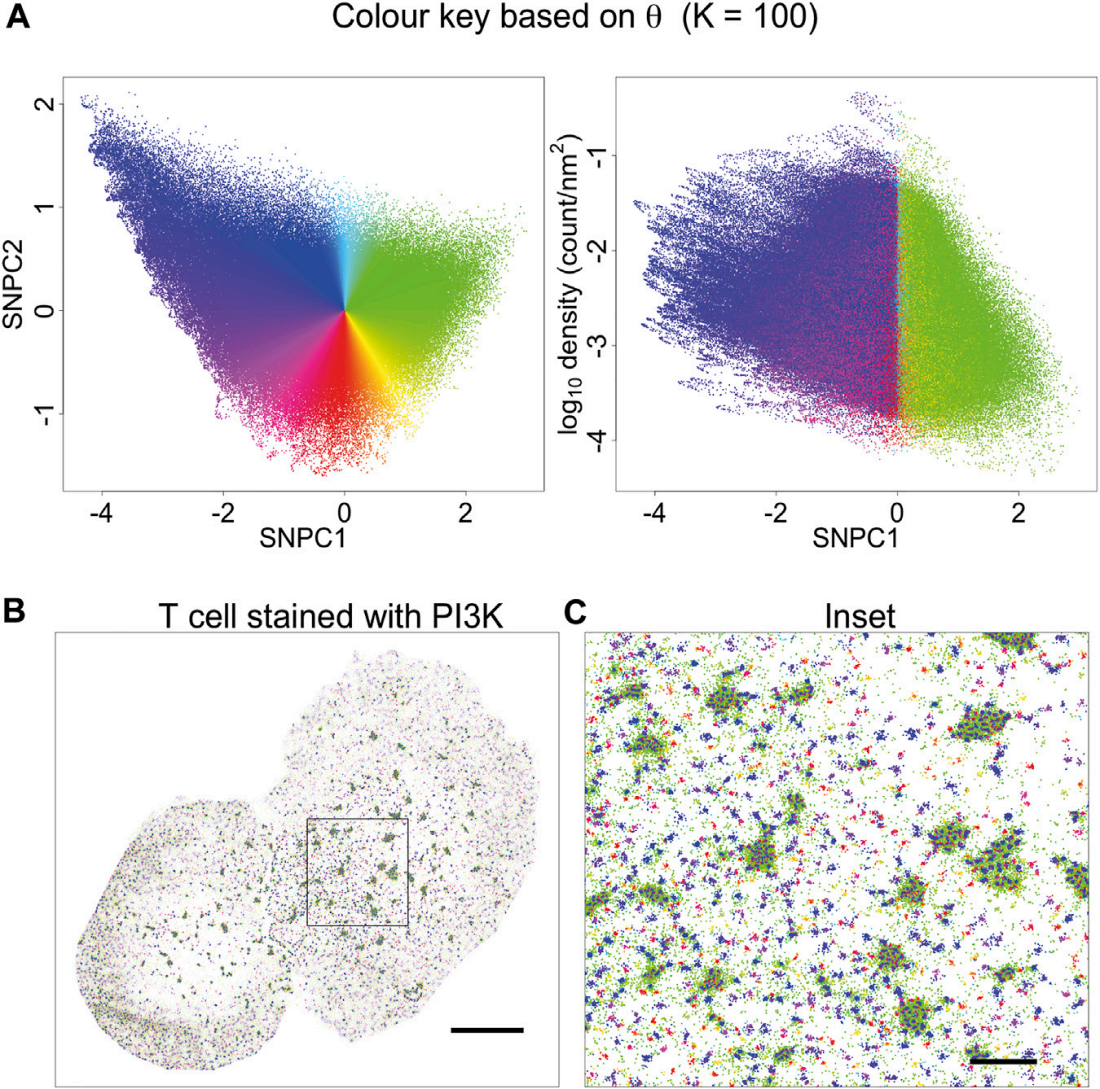
Visualisation based on theta for *K* = 100. **(A)** A novel visualisation is constructed by converting *θ* to hue. **(B)** PAINT image of T cell stained with PI3K probe, and coloured according to **(A)**. This choice of visualisation highlights cluster size (or local aggregation within microclusters) and contrasts aggregation (blue, violet, magenta, red, orange) with segregation (green). **(C)** Inset of **(B)**. This visualisation is limited in that it is only based on a single parameter. Other information is suppressed, for example, segregated points (green) have a different density within or outside microclusters. This indicates that they arise from different processes: they occur in-between tightly packed molecules within microclusters, and as spurious noise outside the microclusters.

Having tested KNA on simulated data we then applied it to a DNA PAINT image of DNA origami rulers. This image is composed of localisations corresponding to a single type of structure randomly scattered over the ROI (Supplementary Figure 8A). As this image has low complexity, the image components can easily be gated in a plot of mean NN distance and SNPC1 (Supplementary Figure 8B). The gated components correspond to specific structures in the image (Supplementary Figures 8C,D). The KNA of an image can also be used to assess parameter choice when clustering, e.g., with DBSCAN (Supplementary Figure 9). Here, this assessment demonstrates that it is hard to find a parameter choice that perfectly captures all localisations belonging to the rulers while rejecting other localisations. Next, KNA is applied to a T cell image stained with a PAINT probe for phosphoinositide 3-kinase (PI3K) binding sites (Figure 4). The structure in the localisation pattern can be visualised by creating a colour key (hue) for each localisation that corresponds to *θ* which is performed at *K* = 100. The colour key is selected such that blue/violet lines up with the largest clusters, and yellow/green lines up with noise localisations, creating an obvious contrast. The visualisation could be further extended by introducing a saturation or value corresponding to *r*, but in practice it became visually confusing.

**FIGURE 5:**
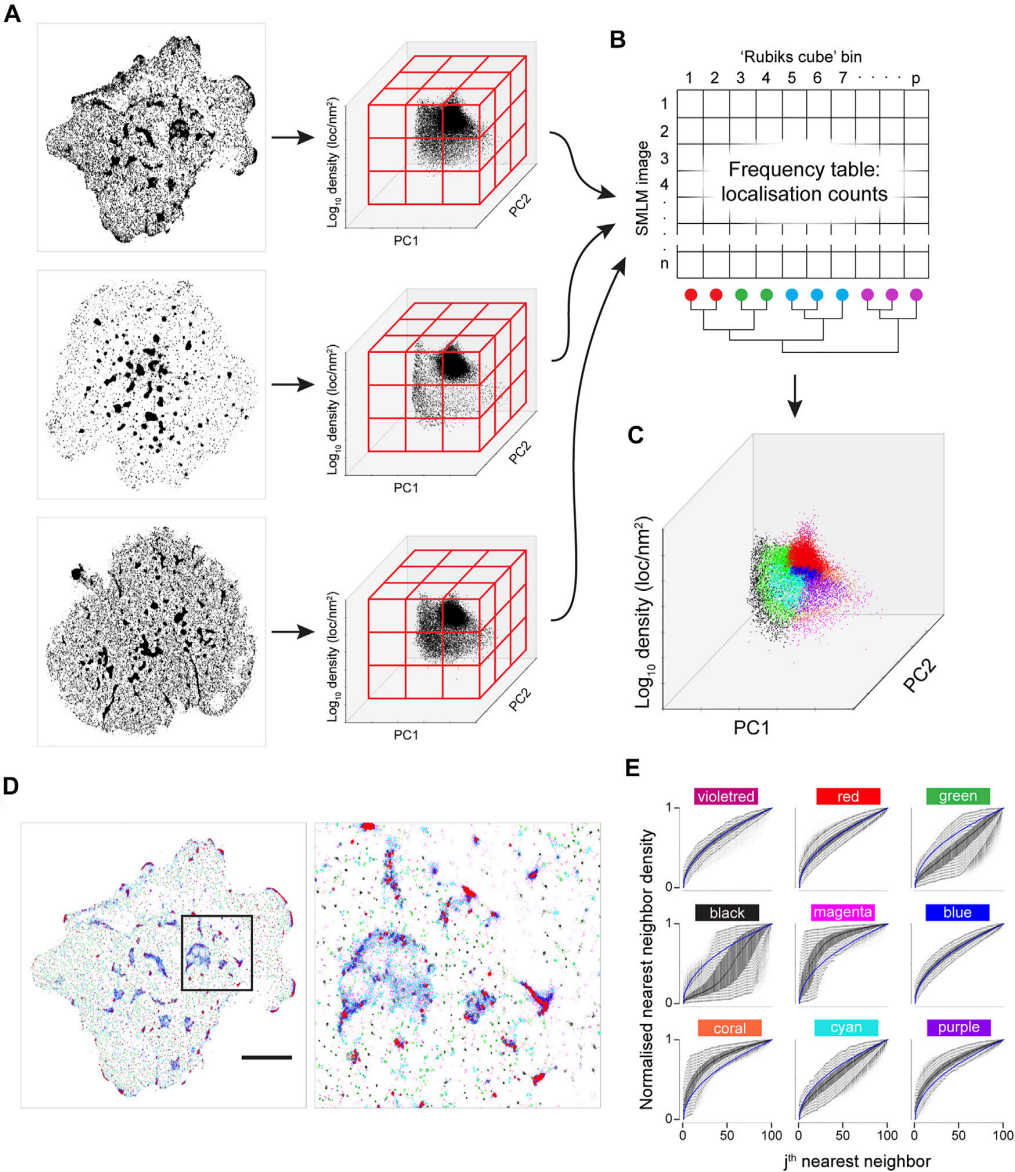
Joint K-neighbourhood analysis over a set of diverse SMLM images. SMLM data are from ILA TCR, first gen CAR and second gen CAR in Jurkat cells activated on supported lipid bilayers containing ICAM-1 and pMHC or CAR ligands. **(A)** Each image is converted into topological coordinates, and the ensuing cloud of points is discretised using a 50 × 50 × 50 grid that is common to all images. Example images of TCR **(top panel)**, first gen CAR **(middle panel)** and second gen CAR **(bottom panel)** are shown. **(B)** The number of localisations in each bin is counted and the grid is unfolded to yield a count vector. The count vector forms a frequency table, where each image is now represented by a row of the frequency table. The bins (columns) are clustered to give localisation groups that have a similar frequency profiles across all images. **(C)** The colour key derived in **(B)** is transferred to the topological coordinate system. **(D)**. The colour key is transferred back to the SMLM image. Scale bar = 5 μm. **(E)** Density-normalized nearest neighbour distances (NND) from the entire dataset for the first *K* = 100 neighbouring localisations for each of the nine spatial organisations arranged in the pattern of the 2-D plot in a. identifies differences in local topology. Colour key as in **(C)**.

**FIGURE 6:**
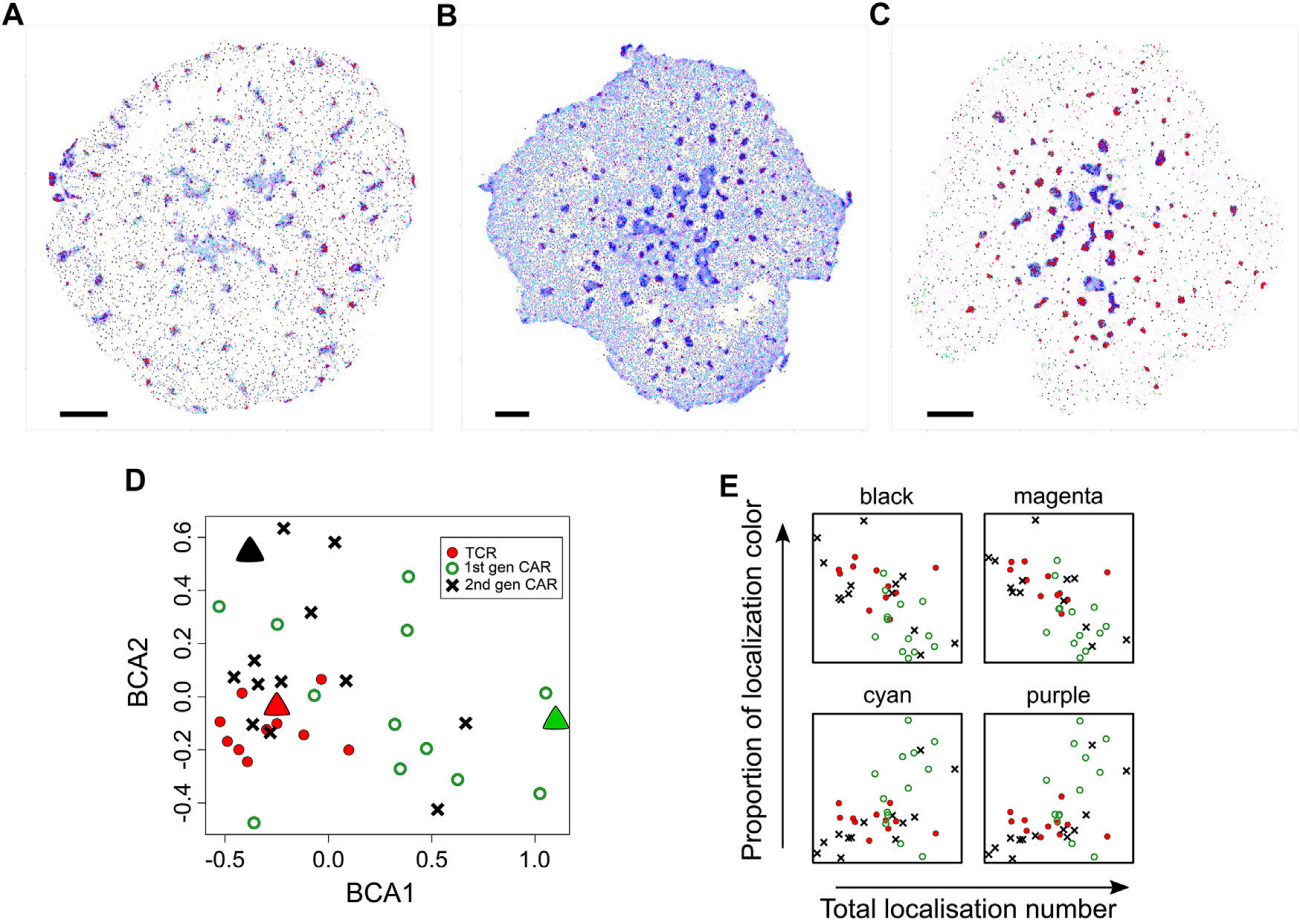
Comparison of diversity in spatial organisation between cells expressing TCR, first generation CAR or second generation CAR. **(A–C)** Representative SMLM images of CD3ζ chain in the TCR-CD3 complex [**(A)**, *n* = 11 images], first generation CAR [**(B)**, *n* = 13 images)] second generation CAR [**(C)**, *n* = 13 images] in Jurkat cells activated on pMHC-containing bilayers. Spatial organisations are colour coded as in Figure 1. Scale bars = 2.5 μm **(D)** Between-component analysis (BCA) of the 9-dimensional frequency table that contains the occupancy of each spatial group as percentages of total localisations. Each symbol represents the total point pattern of TCR (red symbols), first generation CAR (green symbols) and second generation CAR (black symbols) obtained from one SMLM image. Large triangles represent the SMLM images shown in **(A–C)** Adjacent data points indicate that their images have a similar overall spatial composition even though each image is unique. The three data sets are significantly different from each other (Methods) with the TCR exhibiting the least cell-to-cell variability. **(E)** Occupancy of the black, magenta, cyan and purple spatial organisations as a function of expression levels (total localisations) for TCR (red circles), first generation CAR (green open circles) and second generation CAR (black crosses). Other spatial groups showed no correlation with expression levels.

The power of this method is being able to express unique images in a fixed basis which facilitates direct comparison amongst a set of images. This opens up new concepts in SMLM image analysis such as being able to define variance in a set of images, or defining differences between different sets of images. To do this, KNA is performed on each image of the set, and SNPC1, SNPC2 and mean NN distance are retained. A fixed grid is defined in ${\mathbb{R}}^{3}$ (with sets of parallel planes) and the data is binned (Figures 5A,B). The number of points in each bin are counted and the grid is unfolded to yield a frequency vector. Thus each image is now represented by a frequency vector and multivariate statistics can be applied to the set of images. A set of 11T cells images are compared with 13 first generation chimeric antigen receptor (first gen CAR) images and 13 2^nd^ gen CAR images by performing correspondence analysis (CA) on the frequency matrix (columns are first filtered such that all bins are occupied in all images) (Figure 6). While there is overlap between the different sets, they are significantly different from each other. Furthermore, the two CAR sets show a wider variance than the T cell set.

While a visualisation based on KNA was previously developed for individual cells, the frequency table can also be used to develop a joint visualisation for a set of cells (Figures 5B–D). For this, the filtered frequency matrix is first clustered using k-means clustering (*k*_*kmeans*_ = 4). The remaining bins, which are not occupied by all images, become another category. This category is split in two based on density. Finally, frequency clusters that have a large extent in SNPC1 are split into two, with the boundary being SNPC1 = 0 (Figure 3E). In this example, three out of six frequency clusters are split to yield nine frequency clusters, and they were chosen based on visual inspection. The frequency clustering is then converted into a localisation colour key that can be applied to an image. In the k-means step, higher values of *k*_*kmeans*_ were tested (not shown) but this led to a confusing visualisation. The process is summarised graphically in Figure 5. The major intent of this visualisation is to highlight the contrast in global structure between images. For this image set, black, green and cyan generally correspond to nanoclusters with decreasing separation (resp.) to other clusters. Magenta, coral and purple transition from low count nanoclusters to noise localisations, with decreasing separation (resp.) from other clusters. Microclusters are composed of red, blue, cyan and purple localisations, with red patches having the highest density. Finally, violet localisations have a relatively low frequency and often correspond to a very small number of unusually dense nanoclusters. These properties can be gleaned from the n-NND curves (Figure 5E). While the frequency clustering will often be aligned with discrete spatial structures, e.g., nanoclusters, sometimes discrete spatial structures will have memberships of more than one frequency cluster. Given that this method is not intended to be a new spatial clustering method, it is not a necessarily a fault but it does point to future refinements that can be made.

To a rough approximation, KNA will be most sensitive to the regions on the PC axes with the maximum freedom to vary (Supplementary Figure 3). For *K* = 100 this is roughly in the range of 10–70 nearest neighbours and in our T cell receptor dataset this corresponds to nanoclusters and structures within the large microclusters. However longer-range structure can be probed with larger *K* values. Although the local topology description is truncated at *K* = 100, the distribution of single NND values for *K* = 200, 500, partitioned by the nine groups (Supplementary Figure 10), mostly have well defined peaks which are highly reproducible amongst all the T cells. The colours dominating the microclusters consistently have the smallest NNDs. This indicates that the spatial organisations found with *K* = 100 have highly specific relationships to long-range structure. These properties are biologically important because neighbouring spatial organisations are likely to exchange proteins and facilitate protein interactions.

**FIGURE 7:**
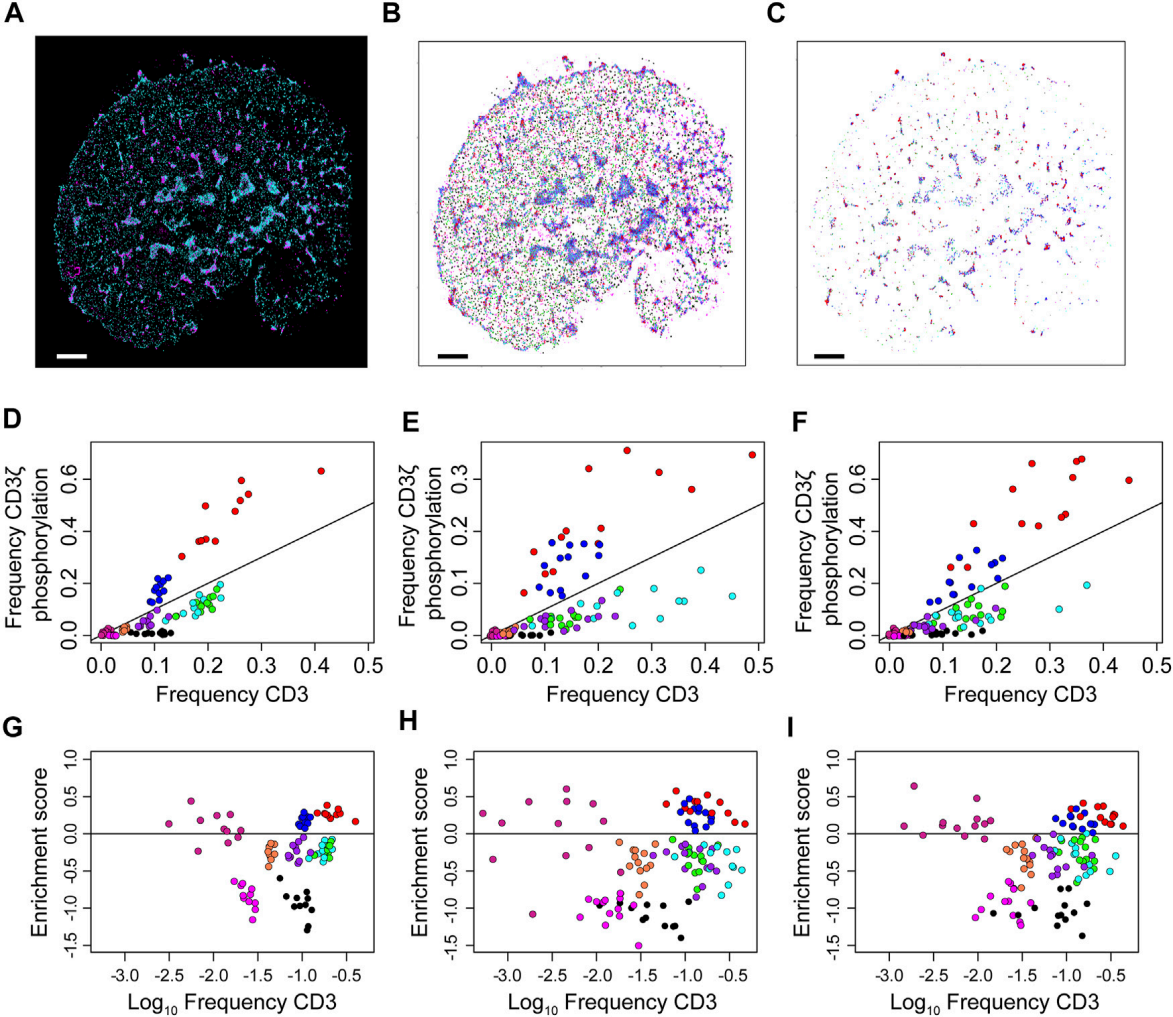
Over- and under-representation of CD3ζ phosphorylation for each of nine co-existing spatial organisations in cells expressing TCR, first generation CAR or second generation CAR. **(A)** Two-colour SMLM images of CD3ζ (cyan) and phosphorylated CD3ζ (pCD3ζ, magenta) of the TCR-CD3 complex in a Jurkat ILA cell activated on a pMHC-decorated bilayer. **(B)** SMLM images of CD3ζ with localisation colour-coded according to the nine spatial groups. **(C)** SMLM images of CD3ζ that are within 10 nm of pCD3ζ localisations. The frequency of these CD3ζ localisations are plotted in **(D–F)**. Colour key as in **(B)**. In **(A–C)**, scales bar = 2.5 μm **(D–F)**. Frequency of CD3ζ phosphorylation as a function of the percentage of total CD3ζ localisations among the nine spatial organisations for TCR **(D)**, first generation CAR **(E)** and second generation CAR **(F)**. Each symbol represents one spatial group from one SMLM image. The black line indicates no preference in phosphorylation; groups above the line are preferably phosphorylated, groups below the line are under-represented in CD3ζ phosphorylation. Note the similarity between the three receptor types. **(G–I)** Phosphorylation enrichment score as a function of CD3ζ occupancy in the nine spatial organisations for TCR **(G)**, first generation CAR **(H)** and second generation CAR **(I)**. Each symbol represents one spatial group from one SMLM image. Symbols above the black horizontal line represent spatial groups that are over-represented in CD3ζ phosphorylation; symbols below the black line represent spatial groups that are under-represented in CD3ζ phosphorylation. The phosphorylation patterns are not significantly different over the three experimental groups (Methods).

Finally, the frequency clusters are used to assess phosphorylation patterns. For each cell, the proportion of co-localised localisations in each frequency cluster is compared to the global proportions of each frequency cluster (Supplementary Figure 11). Here, co-localisation is defined as a CD3 localisation being within 10 nm of a pCD3 localisation. These frequency pairs are used to define a phosphorylation enrichment score, which is the log-ratio of the two frequencies. Scores greater than one indicate an enrichment for phosphorylation while scores less than one indicate a depletion. These results are displayed in Figure 7 and while the three types of cells form markedly different patterns of CD3 clustering, this does not seem to alter the local thresholds for phosphorylation. Enrichment scores are simulated for a random assignment of phosphorylation to each frequency cluster, and the actual enrichment scores are almost always significantly different from random (Supplementary Figure 12). The enrichment scores are re-calculated at multiple co-localisation thresholds up to 500 nm (Supplementary Figure 13). As the co-localisation thresholds increase, the scores approach zero however at different rates for the different frequency clusters. Of particular note are the red and blue localisations, which are consistently the most enriched in phosphorylation across all cell types. The median enrichment score for the red group was 0.29, which means that phosphorylation of red CD3ζ localisations was nearly twice as frequent compared to a hypothetical random phosphorylation event, even though red localisations exhibited a wide variance in number. Although red localisations resided in microclusters, not all spatial groups in microclusters were enriched in phosphorylation suggesting that highly local organisations determined the likelihood of TCR triggering.

## Discussion

The promise of SMLM is to transition away from static biochemical networks, which can be likened to the ingredient list of a recipe, to dynamic spatial signalling networks, i.e., the instructions of a recipe. To do this in a reliable way, it is necessary to move away from the visual inspection of individual cells towards robust statistics over large cohorts of cells. This needs to happen at two levels: picking out the functionally relevant molecular interactions within cells, and quantifying the variance of occurrence across cells (both within and between cell types). To the best of our knowledge, we have presented the first framework which makes it possible to pick out multiscale molecular structures with minimal assumptions, and examine their prevalence across multiple cells with different types of receptors, giving a systematic overview. We have demonstrated that while the three receptor types TCR, first Gen CAR and second gen CAR have general similarities in how they self-arrange, they in fact are subtly distinct from each other which can be attributed to their different structure. The analysis has pooled the information across multiple biological replicates and characterised the variance within the three cell types, which is of key importance in performing reproducible research. We devised a method to characterise the interaction of two molecular species, namely CD3ζ and pCD3ζ, and concluded that the spatial preference of phosphorylation remained constant across the three cell types, despite the differences in receptor spatial composition. Moreover, this analysis can be extended to 1) further types of T cells to further characterise the spatial preference of phosphorylation, 2) other key pairs of molecular species and 3) images of >2 molecular species. Finally, as an outlook, this analysis can be used to build an “atlas”of known cell types (T cell or other interesting cells), to identify commonalities and differences in receptor clustering and also integrate spatial information with other forms of single cell “omics” data. Such an atlas can then be used to classify novel and unknown cell types using machine learning.

## Data Availability

The original contributions presented in the study are included in the article/Supplementary Material, further inquiries can be directed to the corresponding authors.
