# Supplementary material for: K-Neighbourhood Analysis: A Method for Understanding SMLM Images as Compositions of Local Neighbourhoods
Source: Front Bioinform. 2021 Oct 18;1:724127. doi: 10.3389/fbinf.2021.724127 (PMC9581049; doi:10.3389/fbinf.2021.724127)
Supplement: Supplementary file 1 [file DataSheet2.docx]

##################

# K-neighbourhood analysis: a method for understanding SMLM images as compositions of local neighbourhoods.

# Copyright (C) 2021 Kristen Feher

#

# This program is free software: you can redistribute it and/or modify

# it under the terms of the GNU General Public License as published by

# the Free Software Foundation, either version 3 of the License, or

# (at your option) any later version.

#

# This program is distributed in the hope that it will be useful,

# but WITHOUT ANY WARRANTY; without even the implied warranty of

# MERCHANTABILITY or FITNESS FOR A PARTICULAR PURPOSE. See the

# GNU General Public License for more details.

#

# You should have received a copy of the GNU General Public License

# along with this program. If not, see <http://www.gnu.org/licenses/>.

################

# This code will be posted to Github shortly

library(spatstat)

library(pheatmap)

library(ca)

library(dbscan)

library(data.table)

library(RANN)

#########

# The functions that do the work

# calculates spatial neighbourhood PCA that is based on spatially random data, to be applied to the images

make_SNPCA <- function(K, npoints = 100000, winsize = 2, border = 0.5) {

rnd <- coords(rpoispp(npoints, win = owin(c(0, winsize), c(0, winsize))))

w <- rnd[, 1] > border & rnd[, 1] < (winsize-border) & rnd[, 2] > border & rnd[, 2] < (winsize-border)

nn_rnd <- nn2(rnd, rnd[w, ], k = (K+1), eps = 0.01)$nn.dists[, -1]

#

nn_unit_rnd <- t(apply(nn_rnd, 1, function(x) x/max(x)))

pr_rnd <- prcomp(nn_unit_rnd[, 1:(K-1)], center = TRUE, scale. = FALSE)

return(pr_rnd)

}

# Takes a 2-d table of localisations, and augments it with columns corresponding to the K-neighbourhood analysis

make_KNA <- function(D, K, SNPCA, ncomp = 4, saveNN = FALSE, polarcoords = TRUE, hue = TRUE, convert_density = FALSE) {

XY <- colnames(D)

c <- paste0('nn', 1:K)

p <- paste0('PC', 1:ncomp)

NNmean <- 'NNmean'

d <- 'density'

h <- 'hue'

polar <- c('r', 'theta')

D[, (c):=(data.table(nn2(.SD, k = (K+1))[[2]][, -1])), .SDcols= XY]

D[, (NNmean) := rowSums(.SD), .SDcols = (c)]

if (convert_density) {

D[, (d) := log10((K/2 *K^2)/(.SD^2 * pi)), .SDcols = (NNmean)]

}

D[, (NNmean) := log10((NNmean)/K)]

D[, (c) := data.table(t(apply(.SD, 1, function(x) x/max(x)))), .SDcols = (c)]

D[, (p) := data.table(scale(.SD, scale = SNPCA$scale, center = SNPCA$center) %*% SNPCA$rotation[, 1:ncomp]), .SDcols = (c[1:(K-1)])]

if (!saveNN) {

D[, (c) := NULL]

}

if (polarcoords) {

D[, (polar) := data.table(t(apply(.SD, 1, function(x) {z <- complex(real = x[1], imaginary = x[2]); c(Mod(z), floor(Arg(z)/2/pi*360+180))}))), .SDcols = c(p)]

}

if (hue) {

D[, (h) := ((theta-80)%%360)/360]

}

}

# takes a list of KNA data.tables and jointly bins them

make_bins <- function(X, l = 50, C = c('PC1', 'PC2', 'NNmean')) {

lX <- length(X)

range_X <- do.call(rbind, lapply(1:lX, function(k) X[[k]][, lapply(.SD, range), .SDcols = (C)]))

breaks_X <- range_X[, lapply(.SD, function(x) seq(min(x), max(x), length.out = l+1))]

for (j in 1:lX) {

X[[j]][, paste0(C, '_cut') := lapply(1:length(C), function(i) cut(.SD[[i]], breaks_X[[i]], 1:l, include.lowest = TRUE, ordered_result = TRUE)), .SDcols = C]

X[[j]][, binID := do.call(interaction, .SD), .SDcols = paste0(C, '_cut')]

X[[j]][, paste0(C, '_cut') := NULL]

}

}

# takes a list of binned KNA data.tables and makes a frequency table

make_frequency_table <- function(X) {

lX <- length(X)

tab <- lapply(1:lX, function(i) X[[i]][, table(binID)/.N])

tab <- do.call(rbind, tab)

tab <- tab[, colSums(tab) > 0]

return(tab)

}

# takes a list of binned KNA data.tables and the frequency table, and makes frequency cluster

make_freq_cluster <- function(X, tab, nclus = 4, split_clusters = TRUE, NNmean_break = 3) {

lX <- length(X)

w <- apply(tab, 2, function(x) sum(x > 0))

hc <- hclust(dist(t(scale(tab[, w == lX]))), method = 'average')

cut_tree <- cutree(hc, k = nclus)

key_dt <- data.table(

binID = c(colnames(tab[, w == lX]), colnames(tab[, w < lX])),

freq_clus = c(cut_tree, rep(max(cut_tree) + 1, length(which(w < lX))))

)

setkey(key_dt, 'binID')

for (m in 1:lX) {

setkey(X[[m]], 'binID')

X[[m]][key_dt, 'freq_clus' := i.freq_clus, on = 'binID']

}

if (split_clusters) {

for (m in 1:lX) {

X[[m]][freq_clus == nclus + 1 & NNmean > NNmean_break, freq_clus:=nclus + 2]

}

}

}

##################

# pre-process data

# This can be done in your preferred manner, but the output should be a 2-dimensional data.table with X-Y localisation coordinates, with no non-numeric entries, and ideally containing just the outline + contents of cell, i.e. background should have been masked out

# read file

fn <- list.files('../data/')

D <- lapply(fn, function(.x) fread(paste0('../data/', .x)))

ld <- length(D)

# change column names to something more convenient

for (i in 1:ld) {

setnames(D[[i]], c("x [nm]", "y [nm]"), c('X', 'Y'))

}

# filter out non-numeric rows

w <- lapply(D, function(.d) .d[, which(is.na(X) | is.na(Y) | is.nan(X) | is.nan(Y) | is.infinite(X) | is.infinite(Y))])

for (i in 1:ld) {

if (length(w[[i]]) > 0) {

D[[i]] <- D[[i]][-w[[i]]]

}

}

# click out cell outline

P <- lapply(1:ld, function(i) ppp(D[[i]]$X, D[[i]]$Y, window = owin(range(D[[i]]$X), range(D[[i]]$Y))))

# a very primitive gating tool, it does the job ...

# plot(P[[i]][sample(npoints(P[[i]]), 50000), ], pch = 16, cex = 0.2)

# ow1 <- clickpoly(add= TRUE)

# L <- ow1

# save(L, file = 'L.RData')

load('L.RData')

# retrieve only cells within outline

SMLM_images <- lapply(1:ld, function(i) data.table(coords(P[[i]][L])))

# note! the spatstat function 'coords' has renamed the columns as c('x', 'y') regardless of what you have previously named them.

################

# expand x-y table to include KNA values

# The PCA calculation will always be invariant up to a sign change, so it's a good idea to set the seed. Using higher values of K is possible - but the PCA/SVD starts taking a long time to calculate. Eventually this step should be able to be replaced by a formula. If you want to investigate higher values of K, it's better to keep it around 100, do the clustering (see below) then partition NND distributions for K>100 by the clustering (see appendix of paper)

set.seed(42)

SNPCA <- make_SNPCA(K = 100)

# note! the coordinates *must* already be in a data.table format for this to work. Note the different way of calling a function - due to data.table's scoping and copying rules, the following functions updates the data.table it takes as an argument without having to store it in a new variable, i.e. *don't* do 'newDT <- make_KNA(DT, K = 100, SNPCA)' because DT is automatically updated. Equivalently, *don't* do 'DT <- make_KNA(DT, K = 100, SNPCA)'

lX <- length(SMLM_images)

for (i in 1:lX) {

make_KNA(SMLM_images[[i]], K = 100, SNPCA)

}

# example plots

png('cell_K100.png', height = 5000, width = 5000)

i = 20

SMLM_images[[i]][, plot(x, y, pch = 16, cex = 0.7, col = hsv(h = hue))]

rect(20000, 17000, 27000, 24000, lwd = 2)

dev.off()

png('SNPCA_K100.png', height = 450, width = 850)

par(mfrow = c(1, 2), mar = c(6, 6, 6, 1), oma = c(0, 0, 0.3, 0)+0.05)

i = 2

SMLM_images[[i]][, plot(PC1, PC2, pch = 16, cex = 0.2, col = hsv(h = hue), xlab = 'SNPC1', ylab = 'SNPC2', cex.lab = 2, cex.axis = 2)]

SMLM_images[[i]][, plot(PC1, NNmean, pch = 16, cex = 0.2, col = hsv(h = hue), xlab = 'SNPC1', ylab = expression(log["10"]*' mean NND'), cex.lab = 2, cex.axis = 2)]

mtext(expression('Colour key based on '*theta*' K = 100'), outer = TRUE, cex = 2, line = -3)

dev.off()

###################

make_bins(SMLM_images)

tab <- make_frequency_table(SMLM_images)

make_freq_cluster(SMLM_images, tab, nnsum_break = 3)

# visualising

colvec <- c('black', 'red', 'green', 'blue', 'cyan', 'magenta', 'goldenrod', 'coral', 'seagreen', 'purple', 'violetred', 'slateblue', 'orchid')

i = 10

png('freq_clus_cell.png', height = 5000, width = 5000)

SMLM_images[[i]][, plot(x, y, pch = 16, cex = 0.7, col = colvec[freq_clus])]

dev.off()

png('freq_clus_SNPCA.png', height = 5000, width = 5000)

SMLM_images[[i]][, plot(PC1, nnsum, pch = 16, cex = 0.7, col = colvec[freq_clus])]

legend(10, 10, legend = 1:max(SMLM_images[[i]]$freq_clus), fill = colvec[1:max(SMLM_images[[i]]$freq_clus)]) # plot a legend to see which colours correspond to cluster labels for step below

dev.off()

# manually splitting some clusters that have a large extent over PC1 (you will first have to plot NNmean vs. PC1 to select which clusters to split, see example above)

which_clus <- 6

current_names <- unique(unlist(mapply(function(x) unique(x$freq_clus), SMLM_images, SIMPLIFY = FALSE)))

new_clus <- max(current_names) + 1 # safety mechanism to not overwrite an existing frequency cluster

for (m in 1:lX) {

SMLM_images[[m]][freq_clus == which_clus & PC1 > 0, c('freq_clus') := new_clus]

}

# visualising the frequency table

CA <- ca(tab[, colSums(tab) == lX])

plot(CA)

# # if you wish to add an extra colour column for easy plotting, and you can easily repeat to shuffle colours for an aesthetically pleasing result (for-loops not shown)

# i = 1

# SMLM_images[[i]][, freq_colour := colvec[freq_clus]]

# # if you wish to match up KNA with clustering e.g. DBSCAN, and do other stuff with it

# i = 1

# col_names <- c('x', 'y') # localisation coordinate column names

# SMLM_images[[i]][, dbscan_out := dbscan(.SD, eps = 10, minPts = 5)$cluster, .SDcols = col_names]

# # dbscan diagnostic plot

# SMLM_images[[i]][, plot(PC1, NNmean, pch = 16, cex = 0.5, col = factor(dbscan_out == 0))]

# advanced usage: a general method to map an arbitrary grouping of bins back to the localisations

# I generated a completely meaningless factor to demonstrate and the new column will be called 'evenodd'. This can be any kind of vector: factor, integer/real number vector. It will arise when you want to make a custom calculation using the frequency table and want to map it back to the localisations (maybe you didn't like the choice of clustering algorithm I hard coded into make_freq_cluster !)

# my_new_bin_characteristic <- factor(c('even', 'odd')[(1:ncol(tab))%%2+1])

# key_dt <- data.table(

# binID = colnames(tab),

# evenodd = my_new_bin_characteristic

# )

# setkey(key_dt, 'binID')

# for (m in 1:lX) {

# setkey(SMLM_images[[m]], 'binID')

# SMLM_images[[m]][key_dt, 'evenodd' := i.evenodd, on = 'binID']

# }
