# Supplementary material for: K-Neighbourhood Analysis: A Method for Understanding SMLM Images as Compositions of Local Neighbourhoods
Source: Front Bioinform. 2021 Oct 18;1:724127. doi: 10.3389/fbinf.2021.724127 (PMC9581049; doi:10.3389/fbinf.2021.724127)
Supplement: Supplementary file 2 [file DataSheet1.docx]

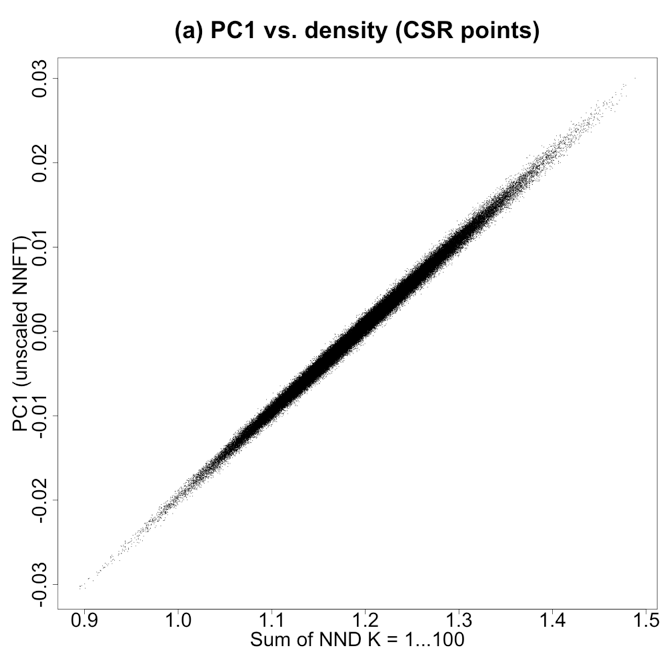

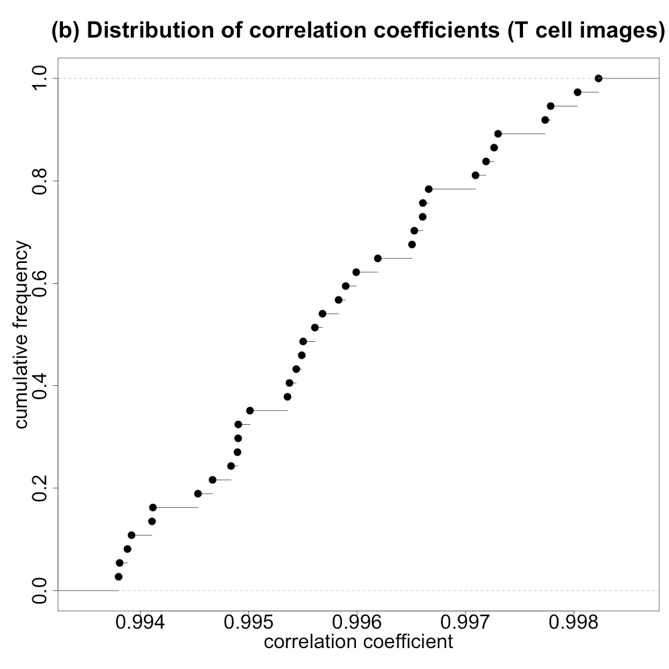


**Supplementary Figure 1. Correlation between localisation density and PC1 of un-normalised NNFT. a** Principal component 1 (PC1) of the unnormalised NNFT of simulated CSR points is plotted against the sum of NNDs for each point (inversely proportional to density). The Pearson correlation coefficient is 0.997. **b** For each T cell image, the PCA of the unscaled NNFT is calculated individually, and the PC1 is compared to the sum of the NNDs. The correlation coefficient is always greater than 0.99. This justifies the partitioning of density from the shape of the NND curves.


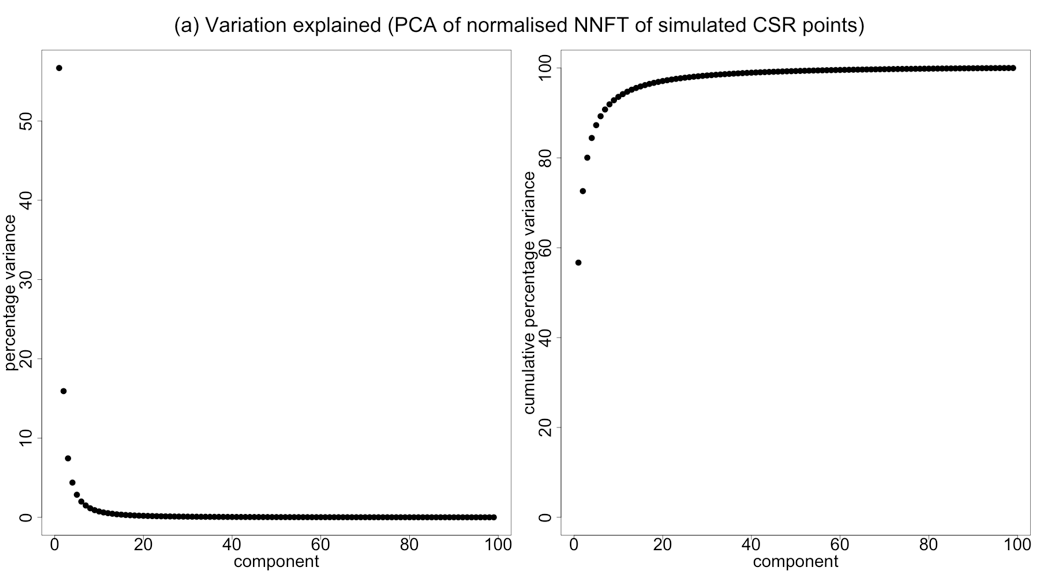


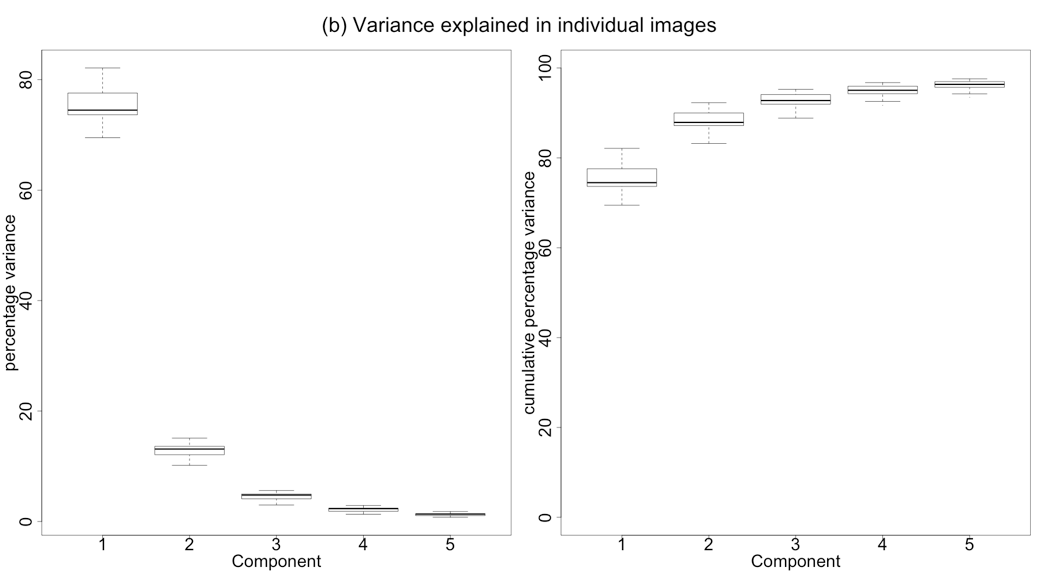


**Supplementary Figure 2. Variance explained in the pre-defined PCA. a** The percentage of variance explained in each principal component arising from the PCA of the row-normalised-NNFT of the simulated CSR points (displayed as a scree plot (left) and cumulative percentage (right)). The first two components explain over 70% of the variance. **b** When the generalised PCA is applied to the RN-NNFTs of the T cell images, the first two components explain over 80% of the variance (displayed as a scree plot (left) and cumulative percentage (right)). Only the first five components are displayed for visual clarity. These plots justify using the first two principal components in the image transformation.


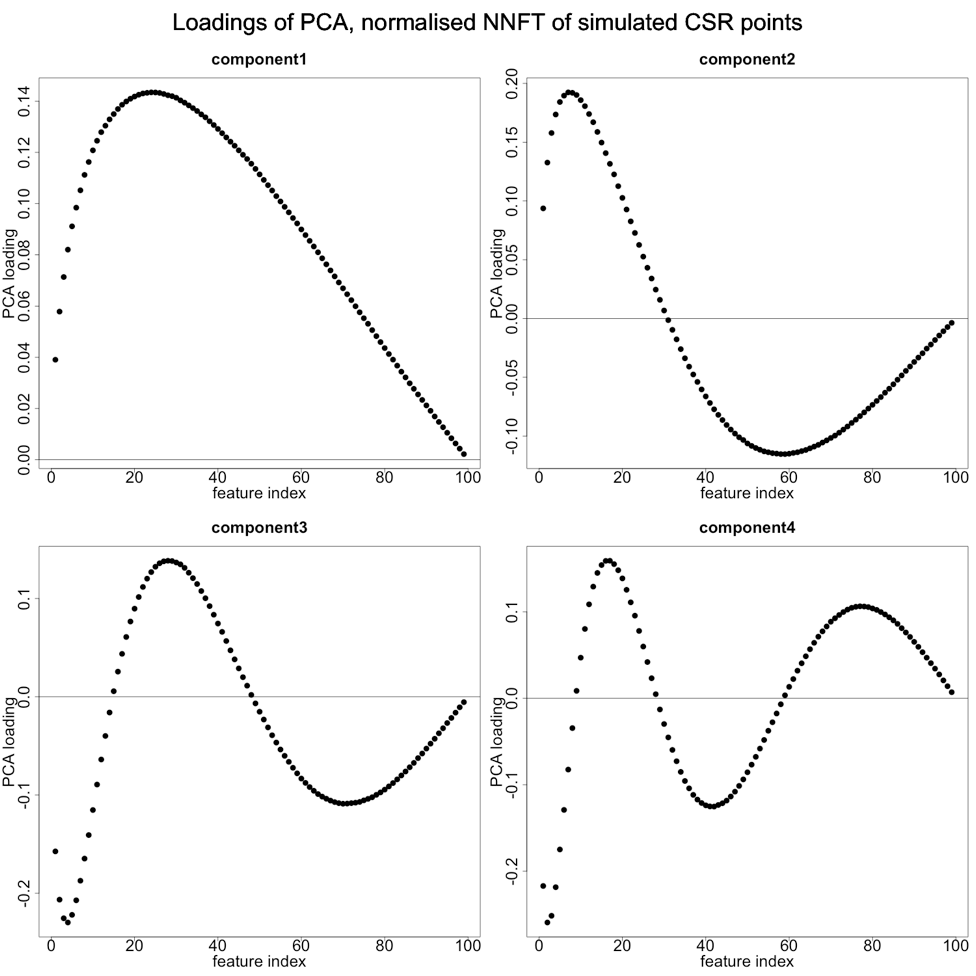


**Supplementary Figure 3. Component loadings arising from PCA of the row-normalised-NNFT of simulated CSR points.** Feature index (x-axis) corresponds to columns of the row-normalised-NNFT. Each plot corresponds to one of the first nine components. In all plots, the PCA loading (y-axis) corresponds to the weight a feature is given in a component. Given that each principal component is a linear combination of the original variables, the loadings correspond to the weight given to each variable in that component. The loadings for the first component are all positive, peaking at *j* = 24. They are close to zero near *j* = 1 and *j* = 100. This is because the NND at *j* = 1 is constrained to be near zero, and the NND at *j* = 100 is constrained to be exactly one. The NND around *j* = 24 has the most freedom to vary. The second component changes sign between *j* = 30 and 31. This reflects the cases when the NND is below average for *j* < 31 and above average for *j* > 30 (and vice versa). In the CSR data, this does not constitute a real pattern, but in the T cell images, corresponds to the clustered vs. segregated localisations. For principal components 3 and higher, the loadings display a ‘periodicity’ of increasing ‘frequency’. (**Supplementary Fig. 4**).


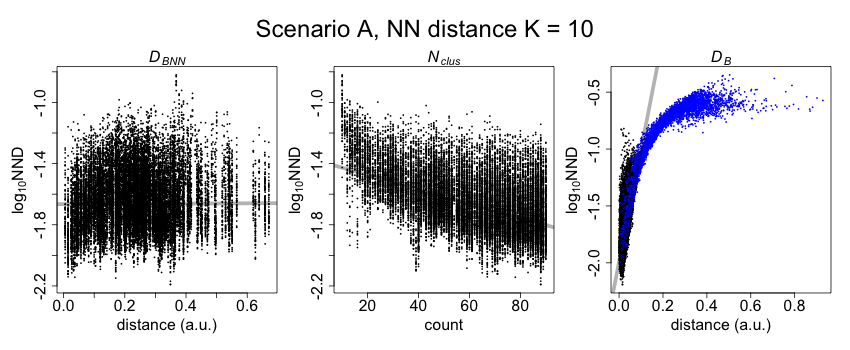


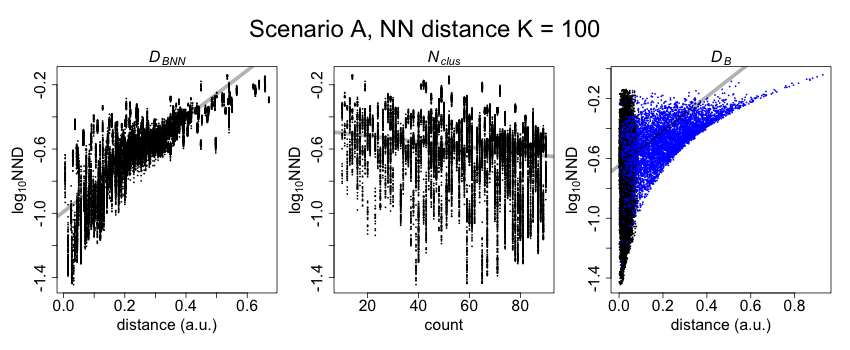


**Supplementary Figure 4. *D_B_, D_BNN_, N_clus_* plotted vs. individual nearest neighbour distances.** At low values of *K* (e.g. *K* = 10), it is possible to differentiate between signal and noise localisations but there is no trend with *D_BNN_*. At high values of *K*, e.g. *K* = 100, there is a trend with *D_BNN_*, but not possible to differentiate between signal and noise localisations. There is no value of *K* which is globally informative of *N_clus_*. This highlights the need to jointly consider multiple values of *K.* Trend lines are fitted with robust linear models and are supplied for visualisation purposes.

**Supplementary Figure 5. Angular position of signal localisations and noise localisations.** Signal and noise localisations occupy distinct areas of the SNPCA.


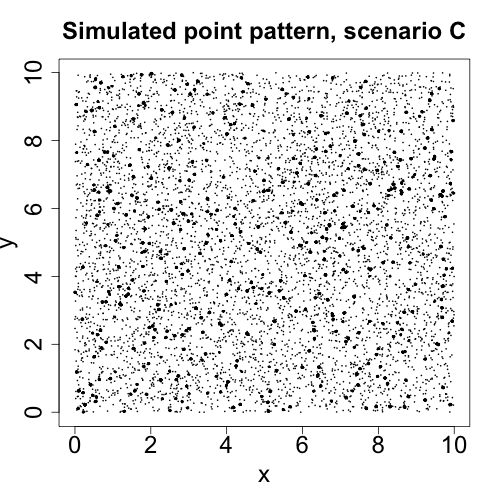

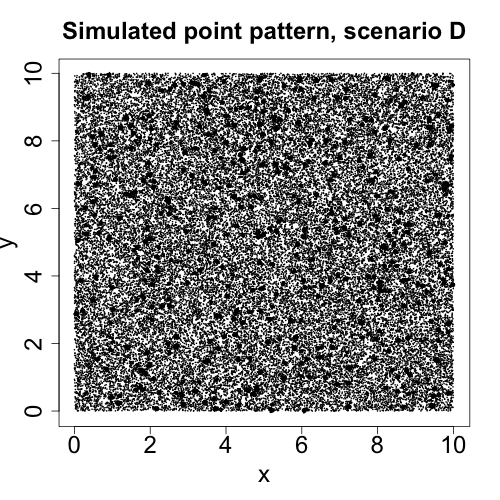


**Supplementary Figure 6. Comparing different noise densities.** Left: Scenario C has low noise density. Binding sites have a Poisson intensity of 5, localisation clusters have a variance of 1.5625e-4 and covariance of 6, number of localisations an integer randomly chosen on the interval [10, 90]. Noise localisations have a Poisson intensity of 50. Right: Scenario D has a high noise density. Simulations details are the same as C except noise localisations have a Poisson intensity of 500.


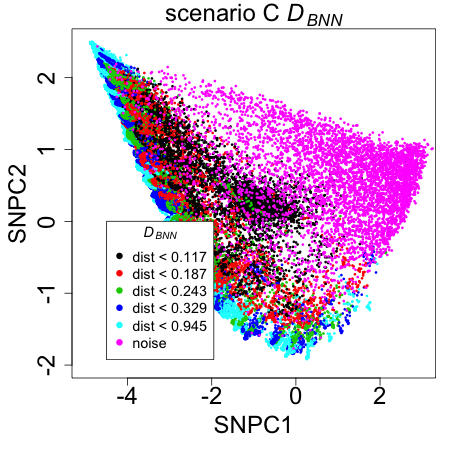

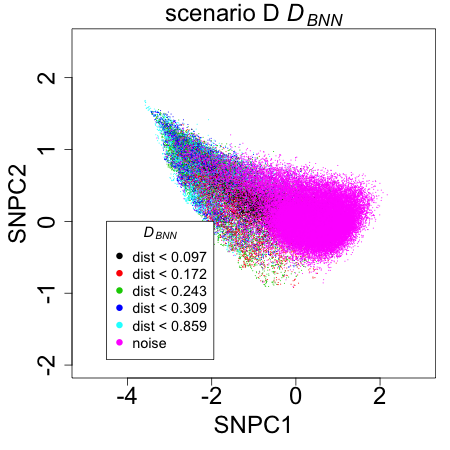


**Supplementary Figure 7**. **Comparing different noise densities.** Top: low density. Noise localisations mostly appear segregated from signal localisations, due to their low density. Bottom: high density. Noise localisations have lower SNPC1 and SNPC2, and often appear to be spatially random due to their high density.


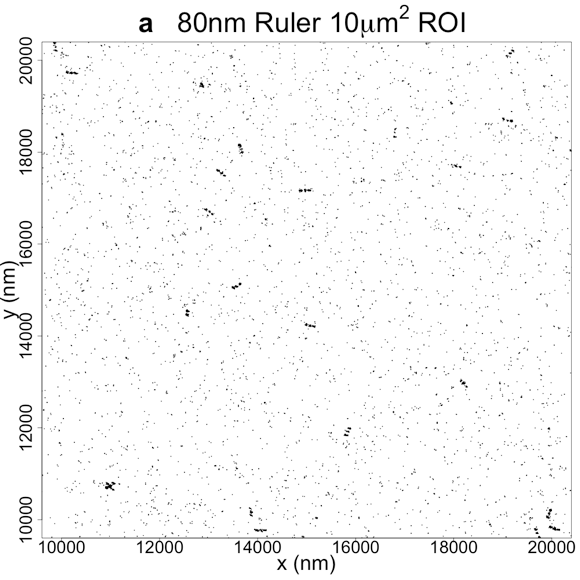

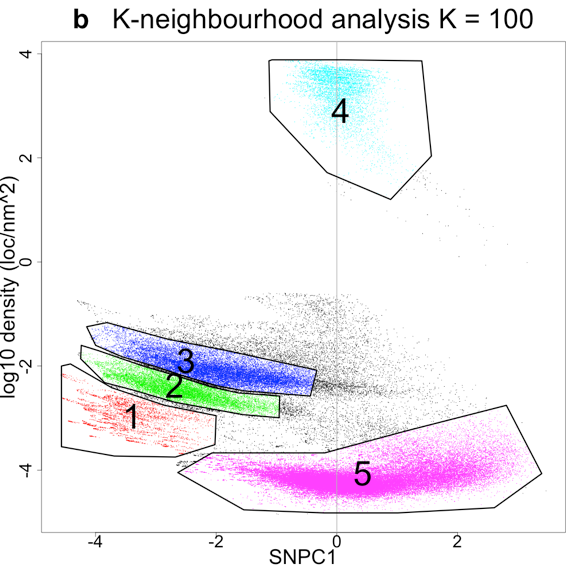


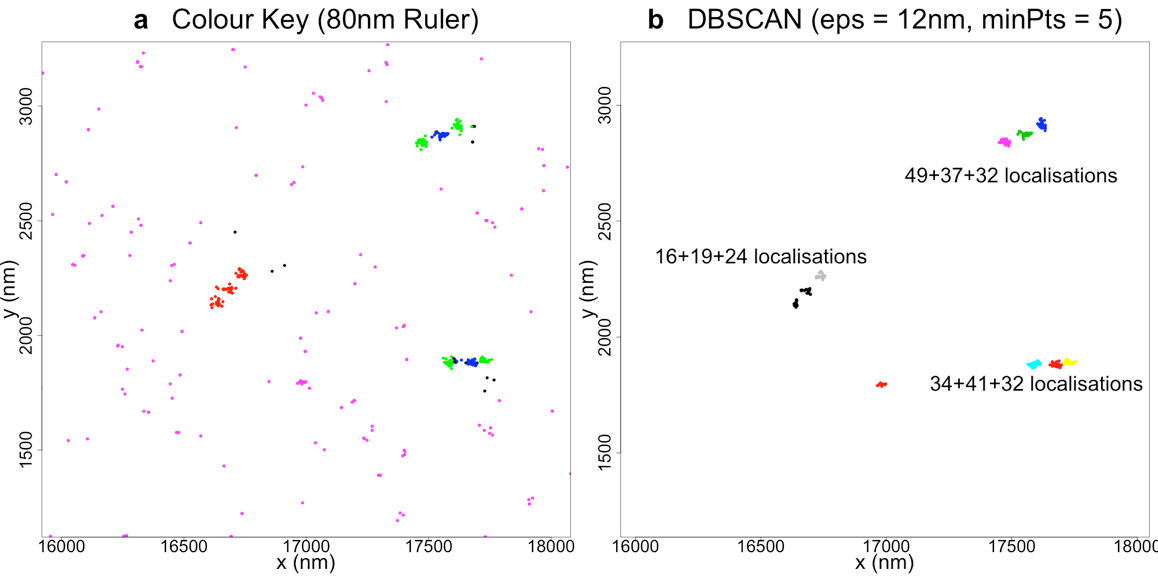


**Supplementary Figure 8 *K*-neighbourhood analysis of an SMLM image. a.** Selected ROI of a DNA PAINT image (total ROI of 40 by 40 micrometre, 8000 frames) of an 80 nm DNA ruler (blue box) with gold nanorod fiducials. **b.** After performing KNA, the density and SNPC1 can be plotted, which is defined as topological space. There is a distinct population structure which is manually gated and localisations in each of the five gates are assigned to a colour key. **c.** A selected ROI is plotted using the colour key defined in (b). Blue, green and red correspond to the rulers. Purple correspond to spurious noise localisations. Cyan corresponds to a fiducials that are present in most frames forming tight, dense localization clusters. **d.** The image is clustered using DBSCAN (eps = 12nm and minPts = 5) to retrieve the individual molecules of the rulers. The number of localisations in each cluster is displayed on the plot.


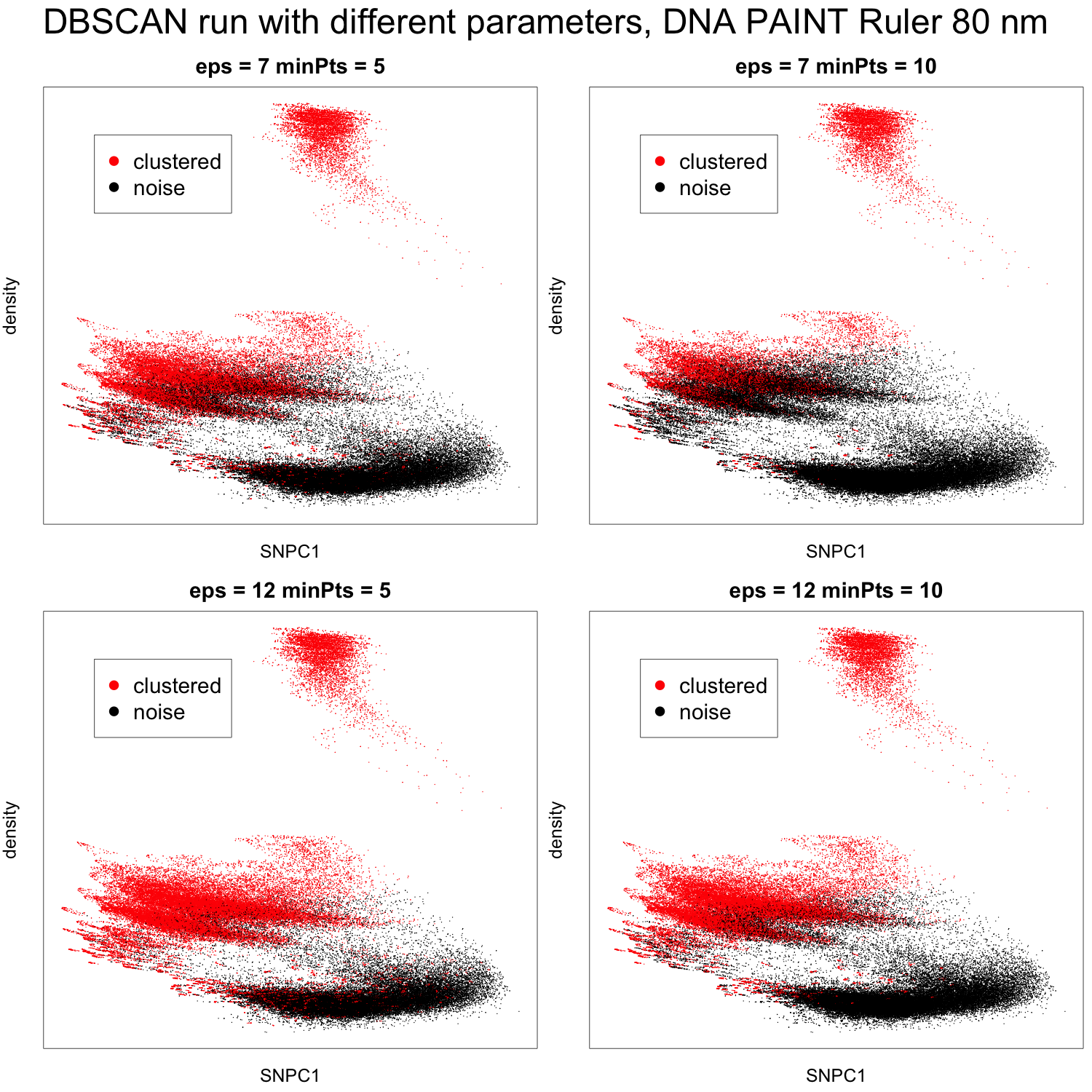


**Supplementary Figure 9. Using KNA as a diagnostic for clustering output.** Continuing Figure 8, density is plotted vs. SNPC1 and localisations are coloured according to whether they have been assigned to a cluster with DBSCAN run at different parameter settings. No single parameter setting can capture all the localisations belonging to the DNA origamis without also picking up spurious clusters in the noise localisations.

**Supplementary Figure 10. The K-neighbourhoods identified with *K* = 100 correspond to specific long-range structure.** **a-b** Long-range structure can be probed by calculating NND for *K* = 200 (**a**) or *K* = 500 (**b**) and partitioning the univariate densities by colour (normalised to have a maximum height of one). The NND distributions indicate how the K-neighbourhoods aggregate (or not) to form multiscale structures. Black and magenta both have a strong peak at a longer distance that other colours, indicating their strong segregation from other structures. Blue and red have strong peaks at shorter distances, indicating their strong participation in microclusters. Green and orange are strongly bimodal, indicating they aggregate with other structures at multiple scales. Biologically speaking, this means that protein clusters are not just defined by area and count of a cluster (e.g. DBSCAN output) but also the structure within and between clusters.

**Supplementary Figure 11.** **Conceptual diagram explaining the calculation of the phosphorylation enrichment score.** **a-b**. First, each CD3ζ localisation was assigned its colour key according to its spatial organisation (here shown as four different colours), following the workflow in **Supplementary Figure 1. a** The frequency of CD3ζ localisations in each spatial group is represented as a frequency vector that was then normalised to sum to one. Next, we asked for each CD3ζ localisation whether it is located within 10 nm of a pCD3ζ localisation (T=True, a) and constructed a normalized frequency vector for co-localised CD3ζ localisation, while maintaining the colour key. **b** A comparison of the two vectors yields the enrichment score, by taking the logratio of the two frequencies for each colour. In this example, red and green localisations have an enrichment score close to zero, indicating no preference for phosphorylation over random selection. Blue CD3ζ localisations are overrepresented in the co-localised CD3ζ frequency vector indicating that blue CD3ζ localisations are preferably phosphorylated. In contrast, purple CD3ζ localisations are underrepresented in the co-localised CD3ζ frequency vector and are therefore deselected for phosphorylation.

**Supplementary Figure 12. Comparison of actual enrichment scores to simulated enrichment scores under the assumption of random phosphorylation.** First, for each cell, phosphorylated events were randomly assigned to each group using a multinomial distribution using cell specific parameters and the corresponding enrichment score was calculated. This simulation is repeated 10000 times and the 5^th^ and 95^th^ percentiles are reported. Finally, the minimum and maximum scores, respectively, over the 37 cells were plotted as horizontal lines. The variance of simulated random enrichment scores is related to the underlying frequency of each group. Note that the variance of the simulated random enrichment scores in certain cases is too small to be visible on the plots (e.g. red and blue groups). The comparison of the actual enrichment scores to the simulated enrichment scores for the TCR (red symbols), 1^st^ generation CAR (green symbols) and 2^nd^ generation CAR (black symbols) demonstrates that there is a strongly non-random spatial component in CD3ζ phosphorylation.

**Supplementary Figure 13. Calculation of Enrichment scores at thresholds of 10, 20, 30 … 500 nm.** Spatial organisations of CD3 are associated with multiscale phosphorylation structure. Black and magenta localisations are strongly deselected for phosphorylation until approximately 400nm, after which they remain weakly deselected, in other words, it is highly unlikely to find phosphorylation within at least 500nm of these localisations. In contrast, cyan and purple are also strongly deselected at 10nm, but the enrichment score rapidly increases and even becomes positive. This means that although any individual receptors corresponding to cyan or purple are unlikely to be phosphorylated, there will often be phosphorylation within 100nm. It’s a similar scenario for green and orange, although without the tendency for scores to become slightly positive. Red and blue localisations’ scores tend to zero but always remain positive, meaning they dominate the phosphorylation response in their local neighbourhoods.
